# Supplementary material for: Color-Tunable Indolizine-Based Fluorophores and Fluorescent pH Sensor
Source: Molecules. 2021 Dec 21;27(1):12. doi: 10.3390/molecules27010012 (PMC8746752; doi:10.3390/molecules27010012)
Supplement: Supplementary file 1 [file molecules-27-00012-s001.zip › molecules-1515856-supplementary.pdf]

Supplementary Materials

Color-Tunable Indolizine-Based Fluorophores and Fluorescent pH Sensor

Taegwan Kim [1] and Jonghoon Kim [1,2,*]

[1] Department of Chemistry, Soongsil University, Seoul 06978, Korea; lever19786@soongsil.ac.kr
[2] Integrative Institute of Basic Science, Soongsil University, Seoul 06978, Korea
* Correspondence: jhkim19@ssu.ac.kr

Absorption and Emission Spectra of Compounds

Blue line: absorption spectra **Red line: emission spectra**

6

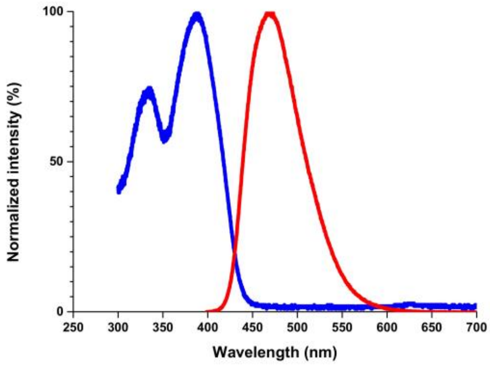

7

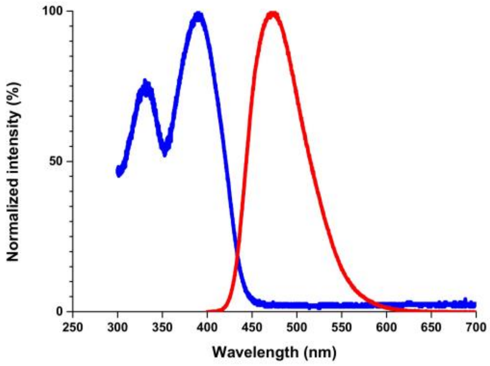

8

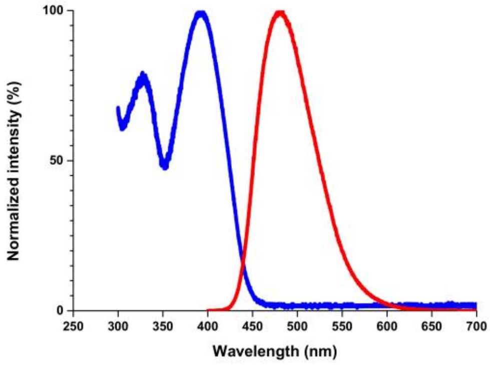

9

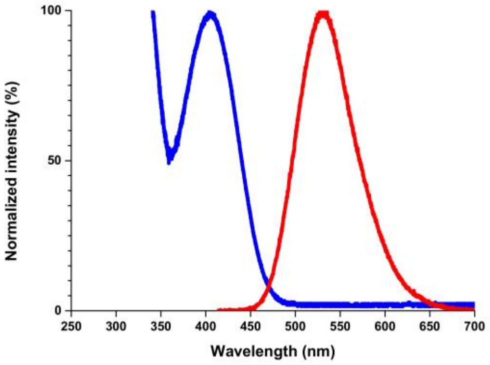

9 / pH 3.0

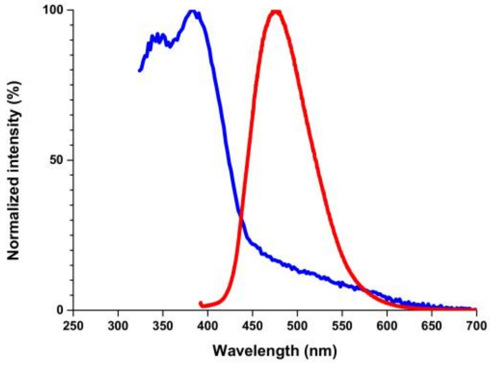

10

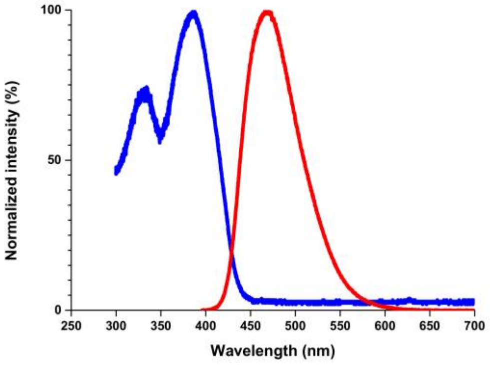

11

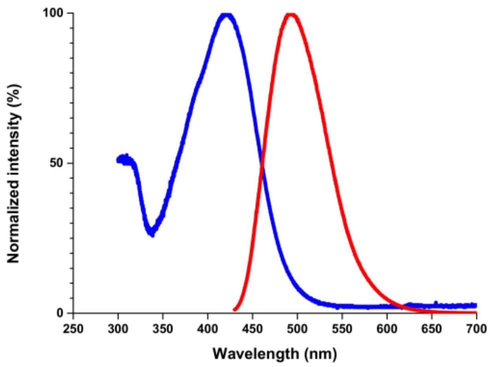

12

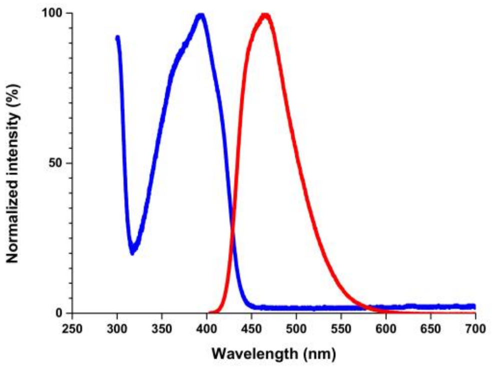

12 / pH 3.0

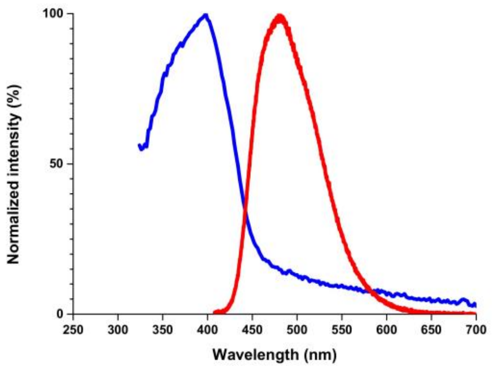

13

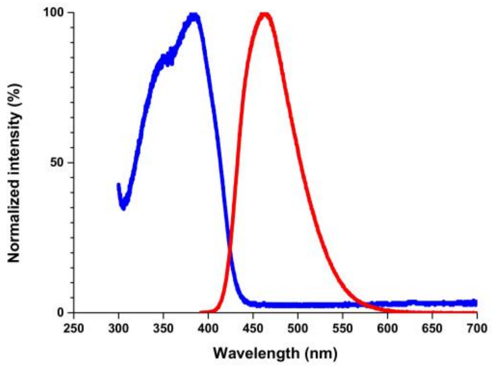

14

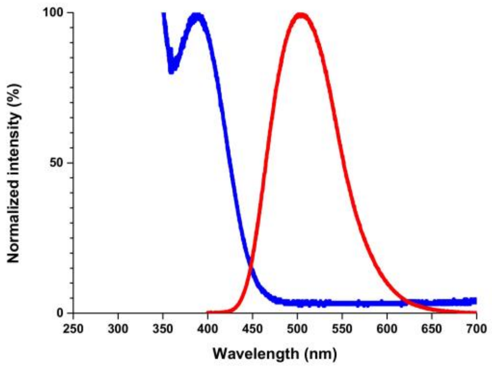

16

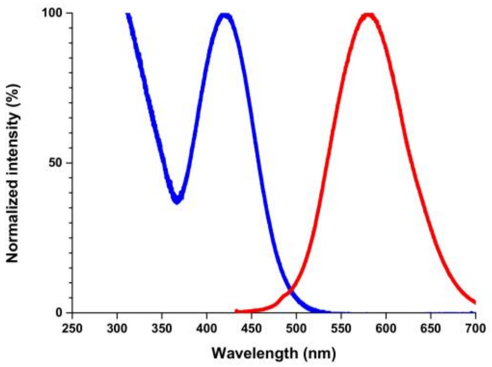

16 / pH 3.0

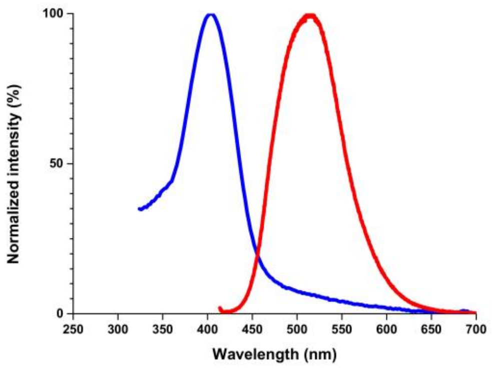

17

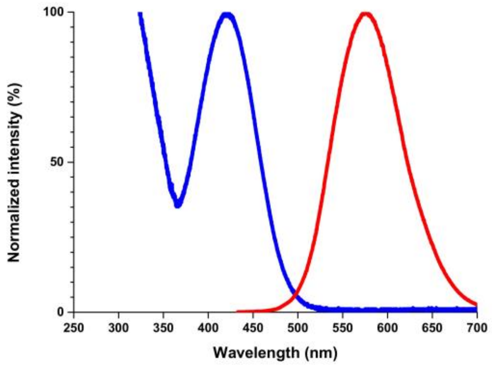

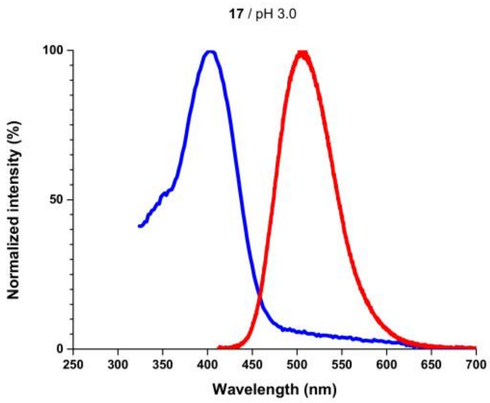

17 / pH 3.0
100
50
0
Normalized intensity (%)
250
300
350
400
450
500
550
600
650
700
Wavelength (nm)

^{1}H and ^{13}C NMR Spectra

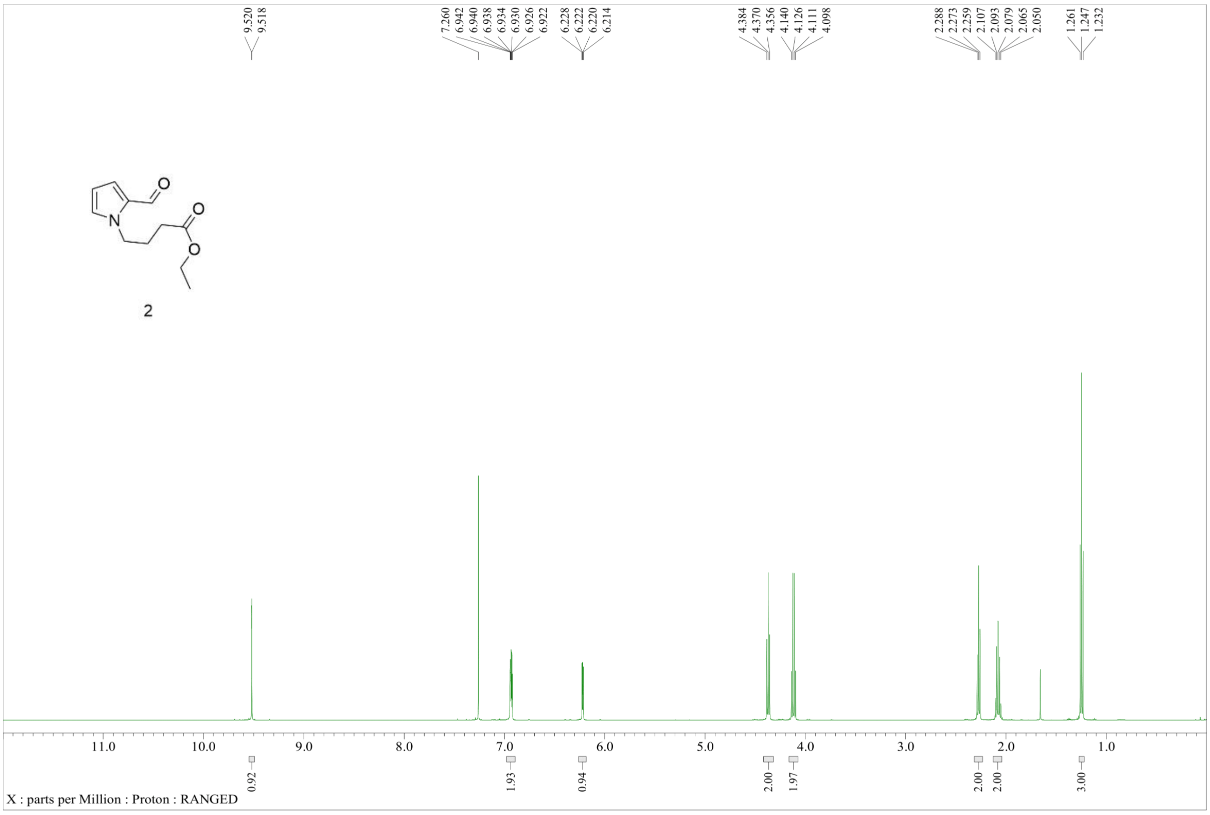

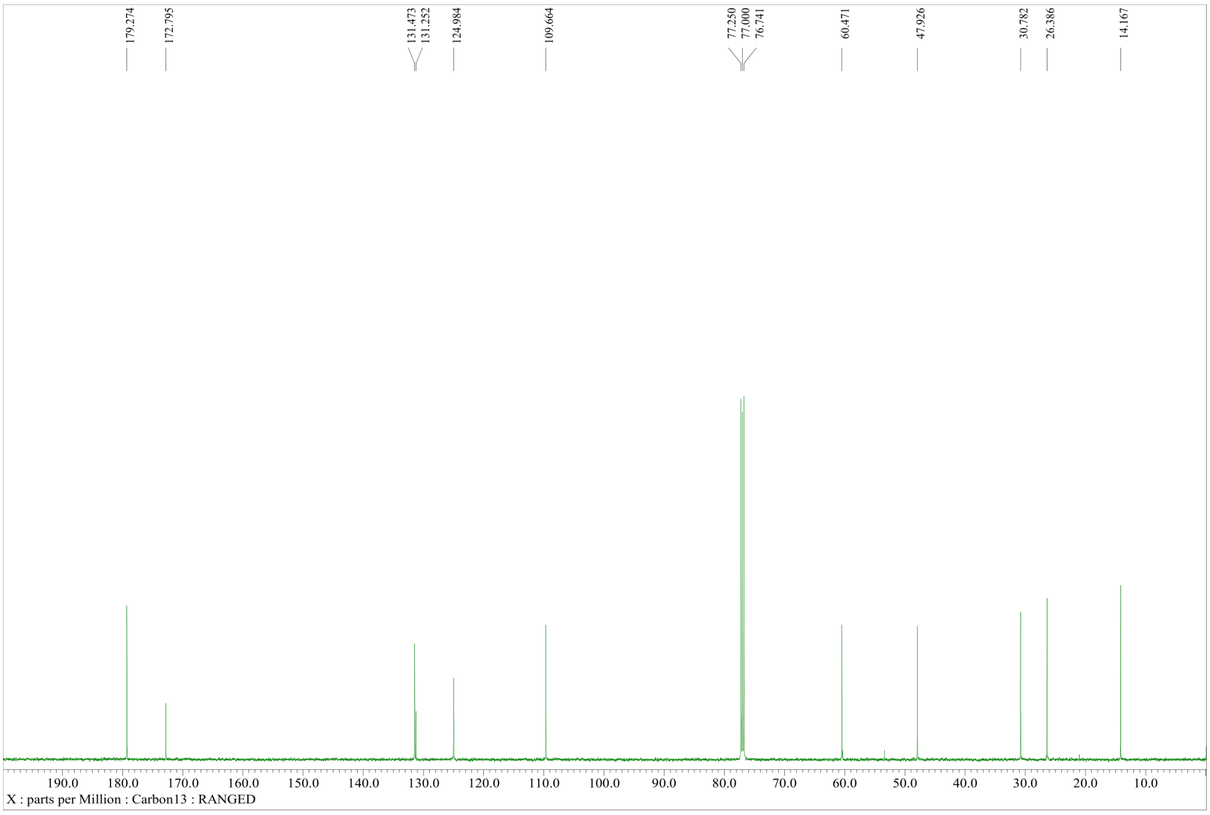

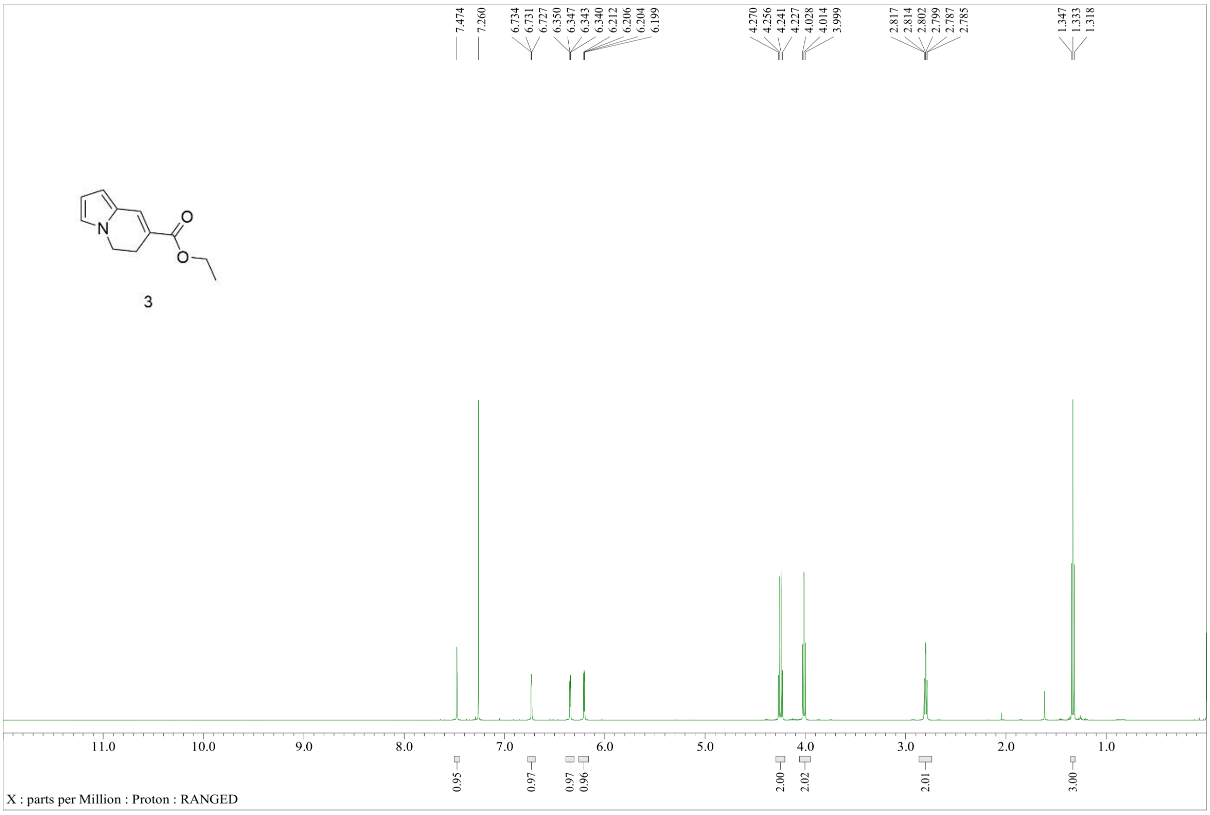
7.474
7.260
6.734
6.731
6.727
6.350
6.347
6.343
6.340
6.212
6.206
6.204
6.199
4.270
4.256
4.241
4.227
4.028
4.014
3.999
2.817
2.814
2.802
2.799
2.787
2.785
1.347
1.333
1.318
3
0.95
0.97
0.97
0.96
2.00
2.02
2.01
3.00
X : parts per Million : Proton : RANGED

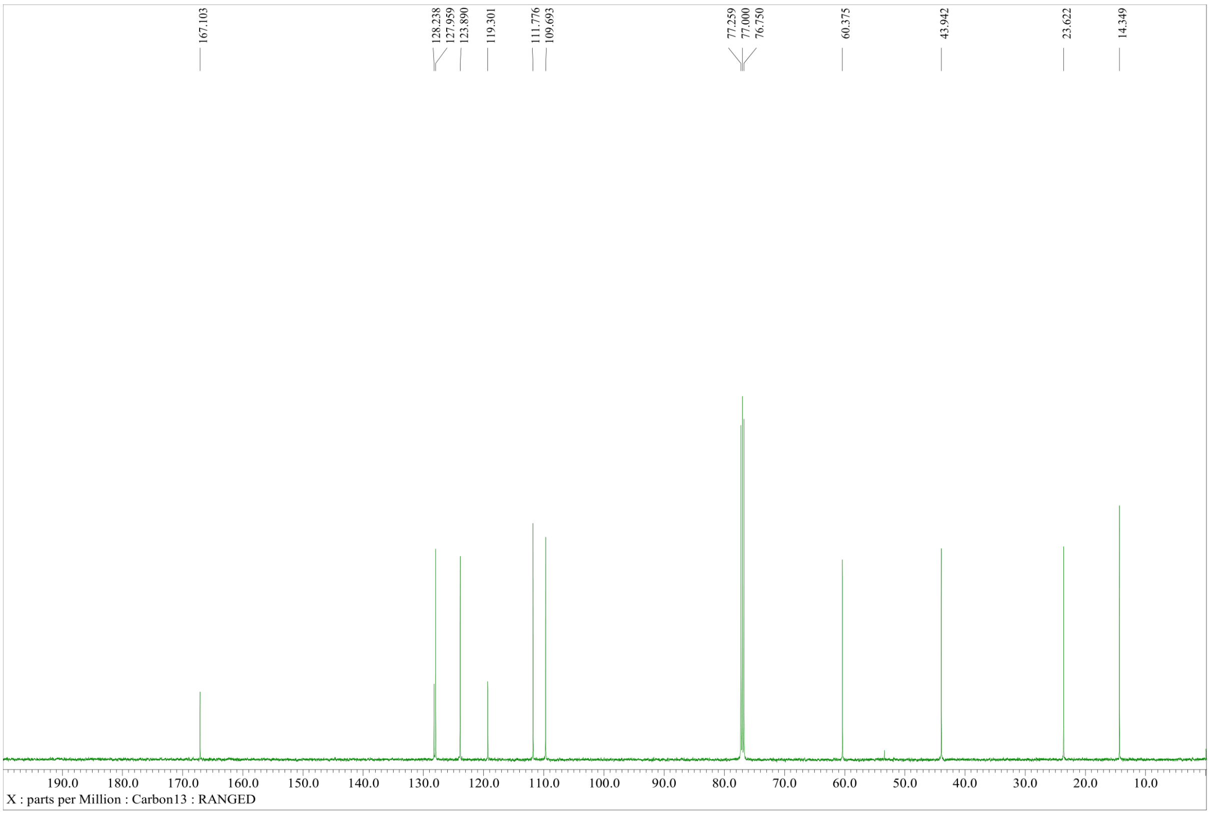
167.103
128.238
127.959
123.890
119.301
111.776
109.693
77.259
77.000
76.750
60.375
43.942
23.622
14.349
X : parts per Million : Carbon13 : RANGED

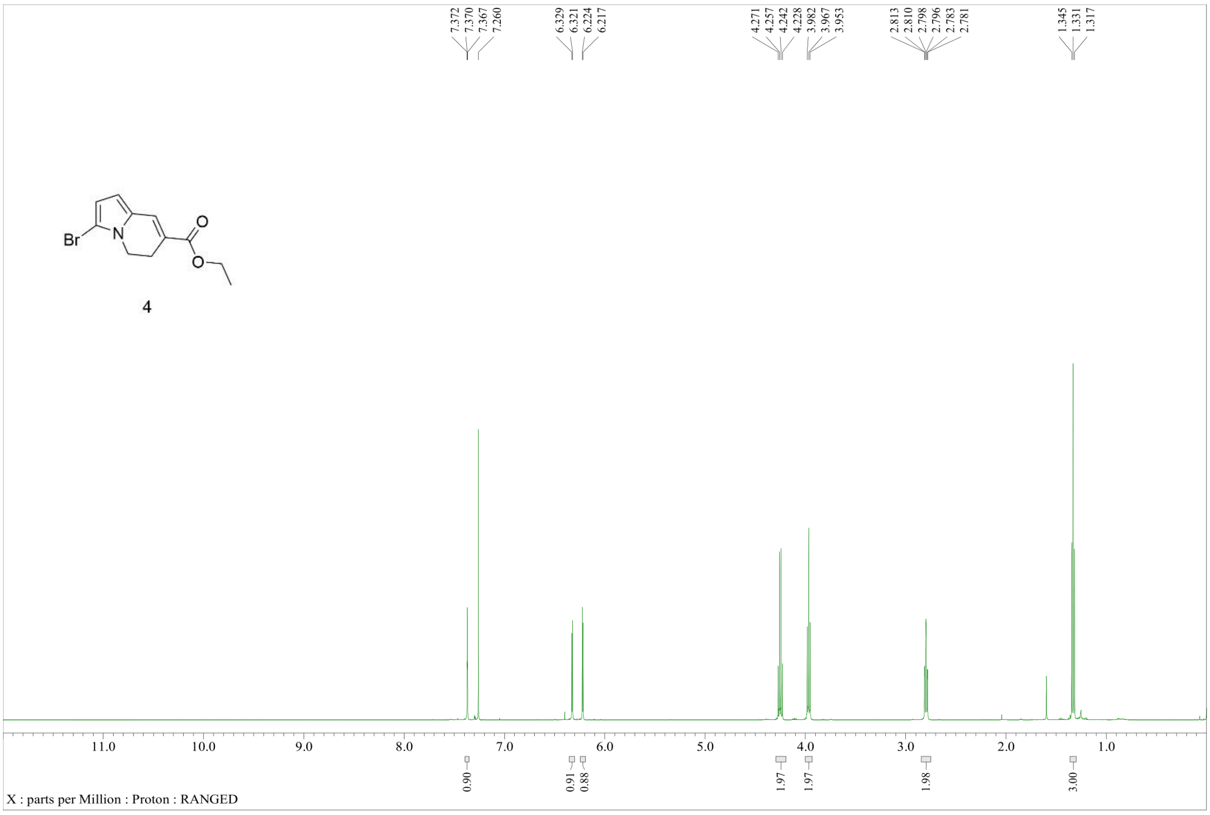
7.372
7.370
7.367
7.260
6.329
6.321
6.224
6.217
4.271
4.257
4.242
4.228
3.982
3.967
3.953
2.813
2.810
2.798
2.796
2.783
2.781
1.345
1.331
1.317
Br
N
O
O
4
11.0
10.0
9.0
8.0
7.0
6.0
5.0
4.0
3.0
2.0
1.0
0.90
0.91
0.88
1.97
1.97
1.98
3.00
X : parts per Million : Proton : RANGED

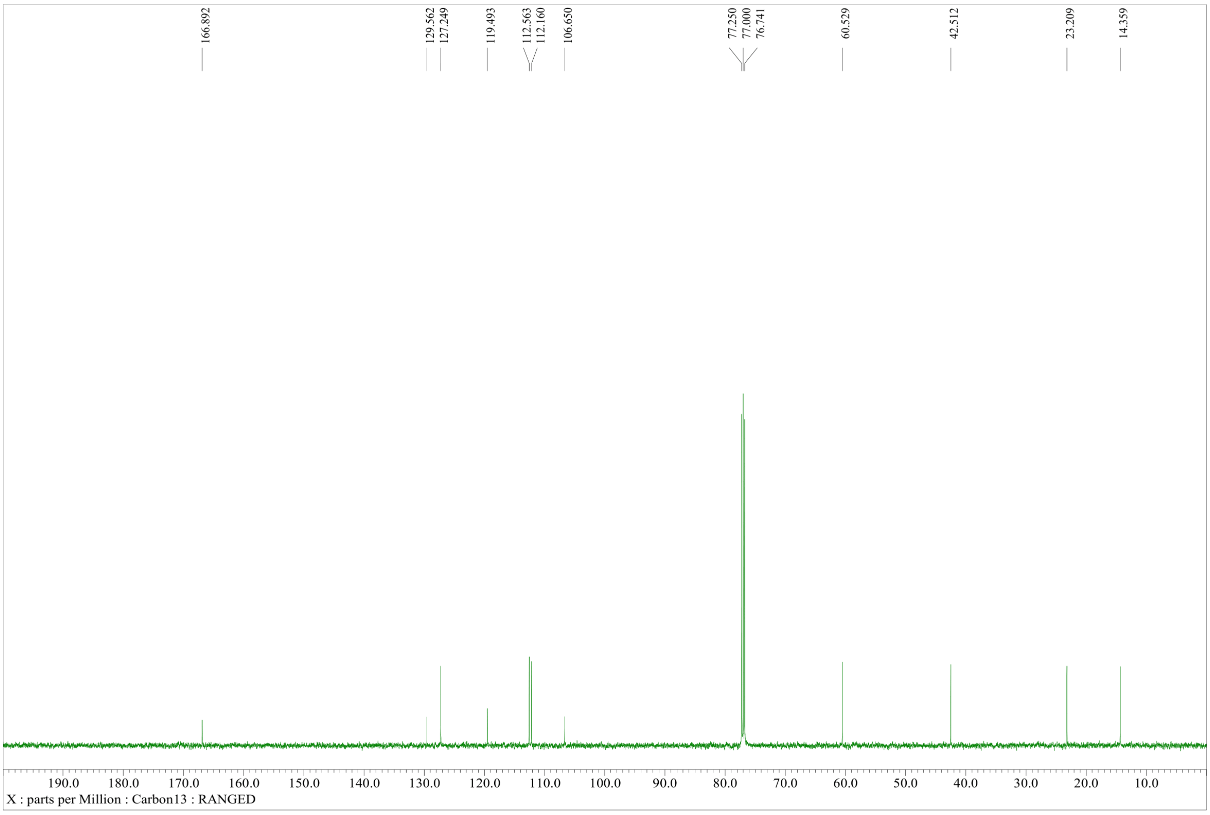
166.892
129.562
127.249
119.493
112.563
112.160
106.650
77.250
77.000
76.741
60.529
42.512
23.209
14.359
190.0
180.0
170.0
160.0
150.0
140.0
130.0
120.0
110.0
100.0
90.0
80.0
70.0
60.0
50.0
40.0
30.0
20.0
10.0
X : parts per Million : Carbon13 : RANGED

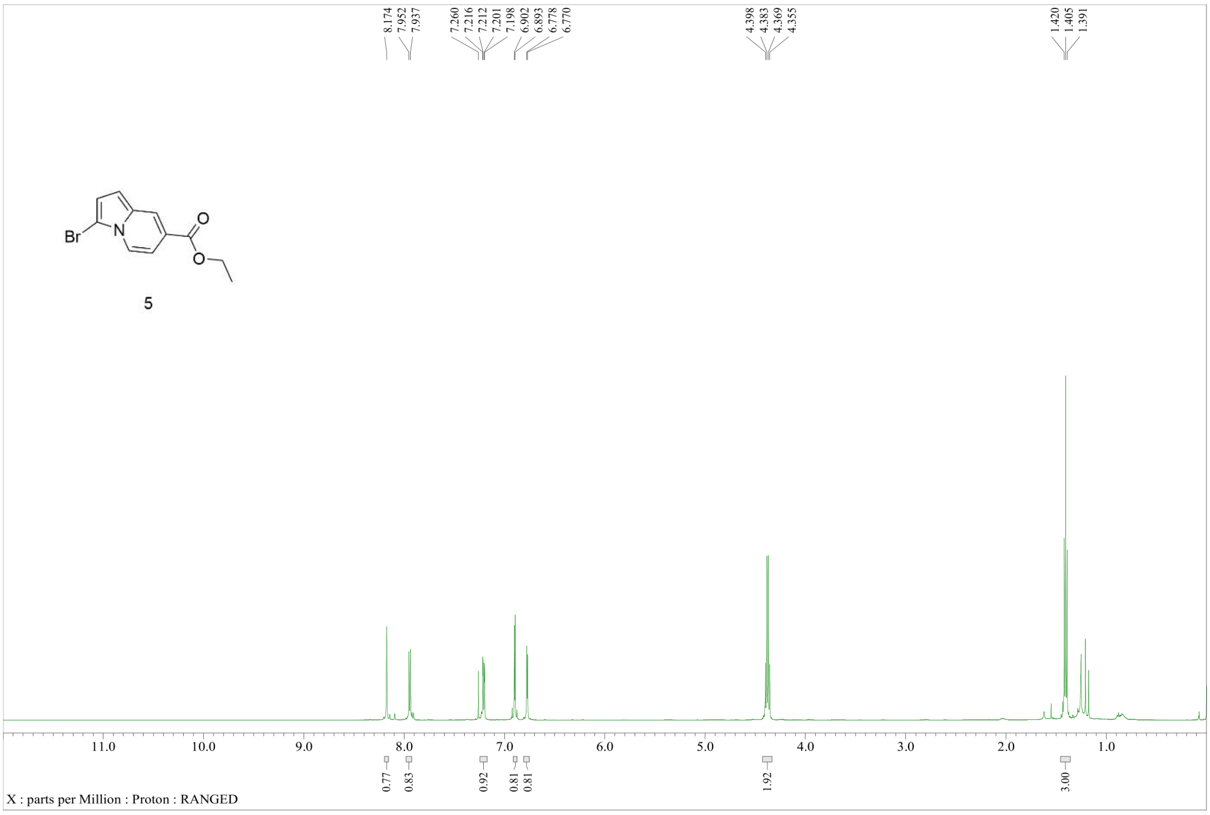
8.174
7.952
7.937
7.260
7.216
7.212
7.201
7.198
6.902
6.893
6.778
6.770
4.398
4.383
4.369
4.355
1.420
1.405
1.391
Br
N
O
O
5
0.77
0.83
0.92
0.81
0.81
1.92
3.00
X : parts per Million : Proton : RANGED

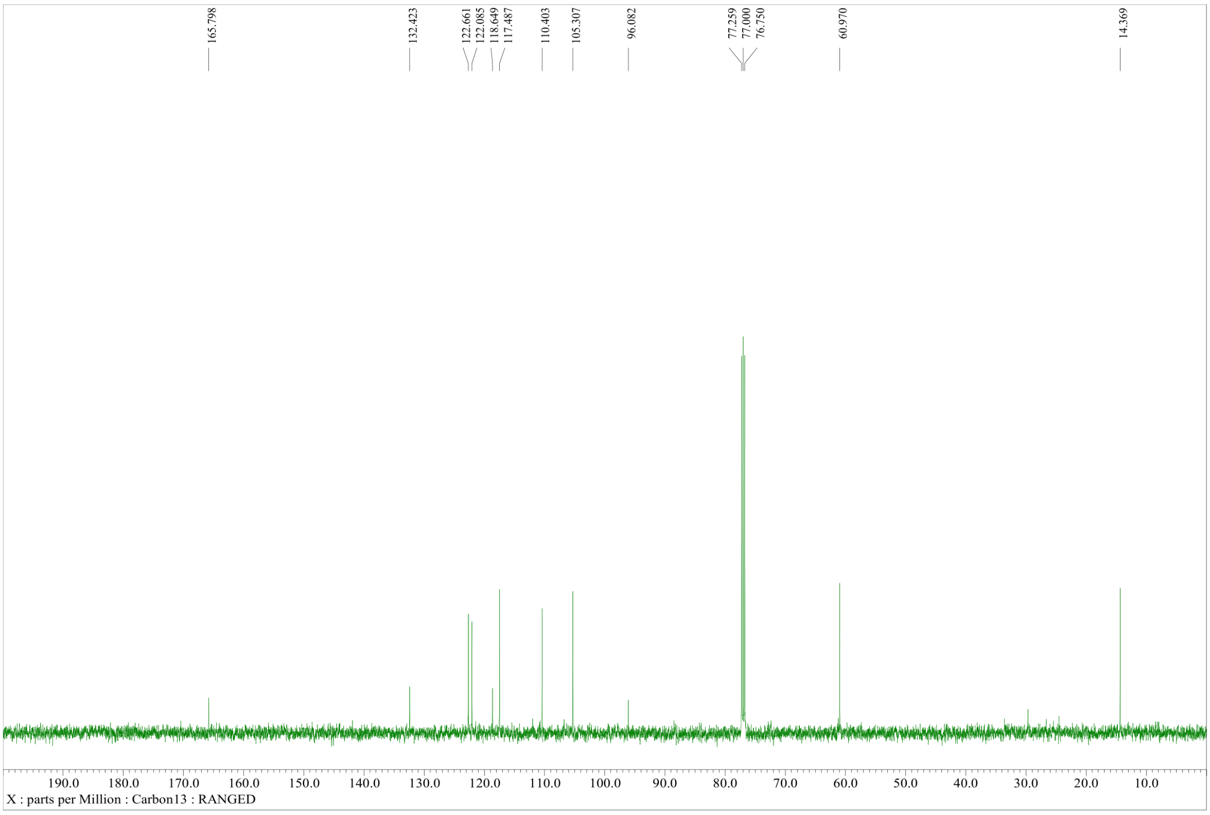
165.798
132.423
122.661
122.085
118.649
117.487
110.403
105.307
96.082
77.259
77.000
76.750
60.970
14.369
X : parts per Million : Carbon13 : RANGED

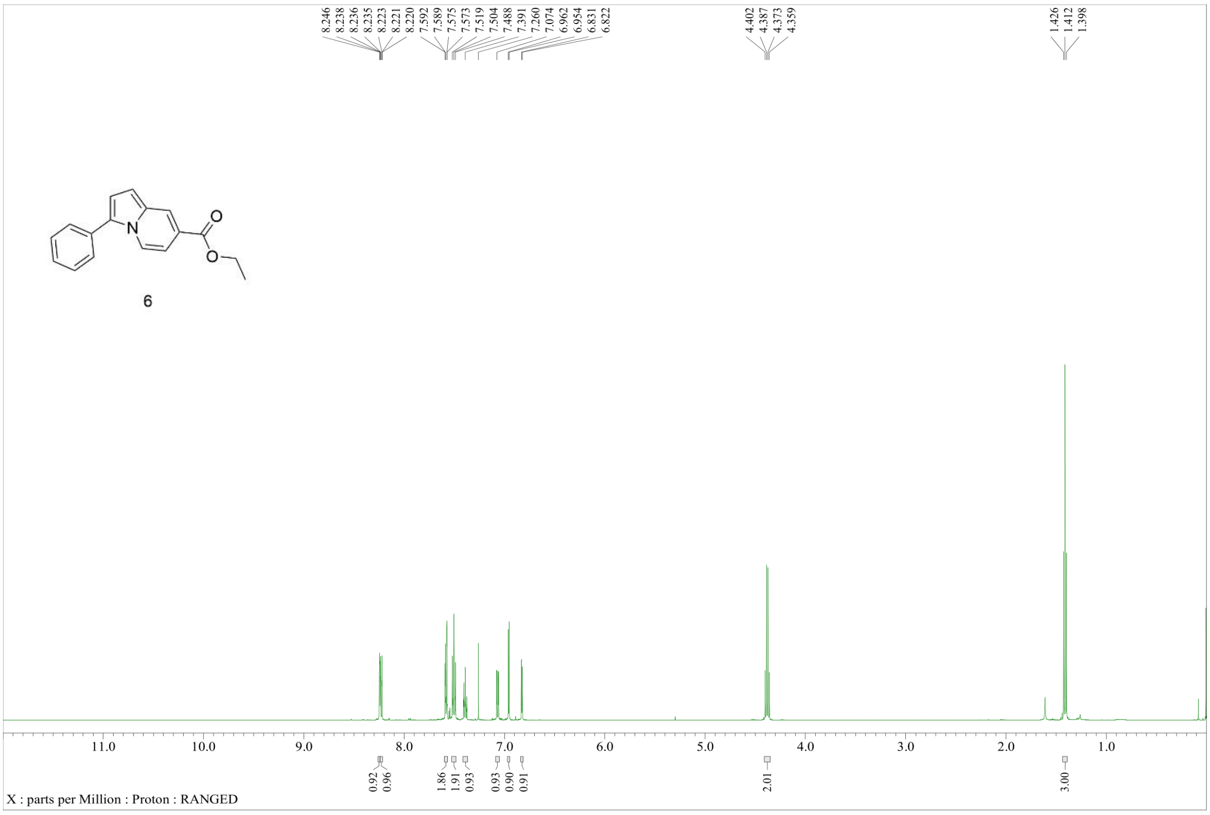
8.246
8.238
8.236
8.235
8.223
8.221
8.220
7.592
7.589
7.575
7.573
7.519
7.504
7.488
7.391
7.260
7.074
6.962
6.954
6.831
6.822
4.402
4.387
4.373
4.359
1.426
1.412
1.398
6
11.0
10.0
9.0
8.0
7.0
6.0
5.0
4.0
3.0
2.0
1.0
0.92
0.96
1.86
1.91
0.93
0.93
0.90
0.91
2.01
3.00
X : parts per Million : Proton : RANGED

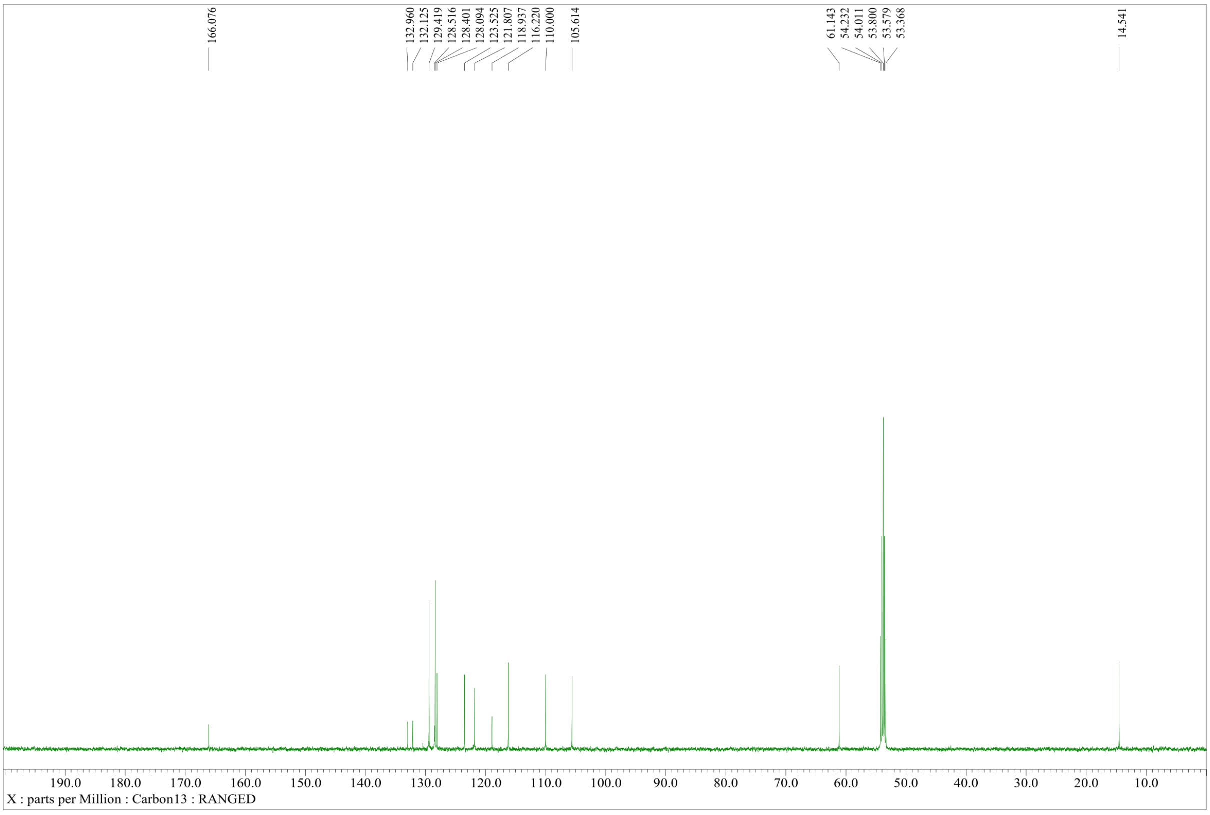
166.076
132.960
132.125
129.419
128.516
128.401
128.094
123.525
121.807
118.937
116.220
110.000
105.614
61.143
54.232
54.011
53.800
53.579
53.368
14.541
190.0
180.0
170.0
160.0
150.0
140.0
130.0
120.0
110.0
100.0
90.0
80.0
70.0
60.0
50.0
40.0
30.0
20.0
10.0
X : parts per Million : Carbon13 : RANGED

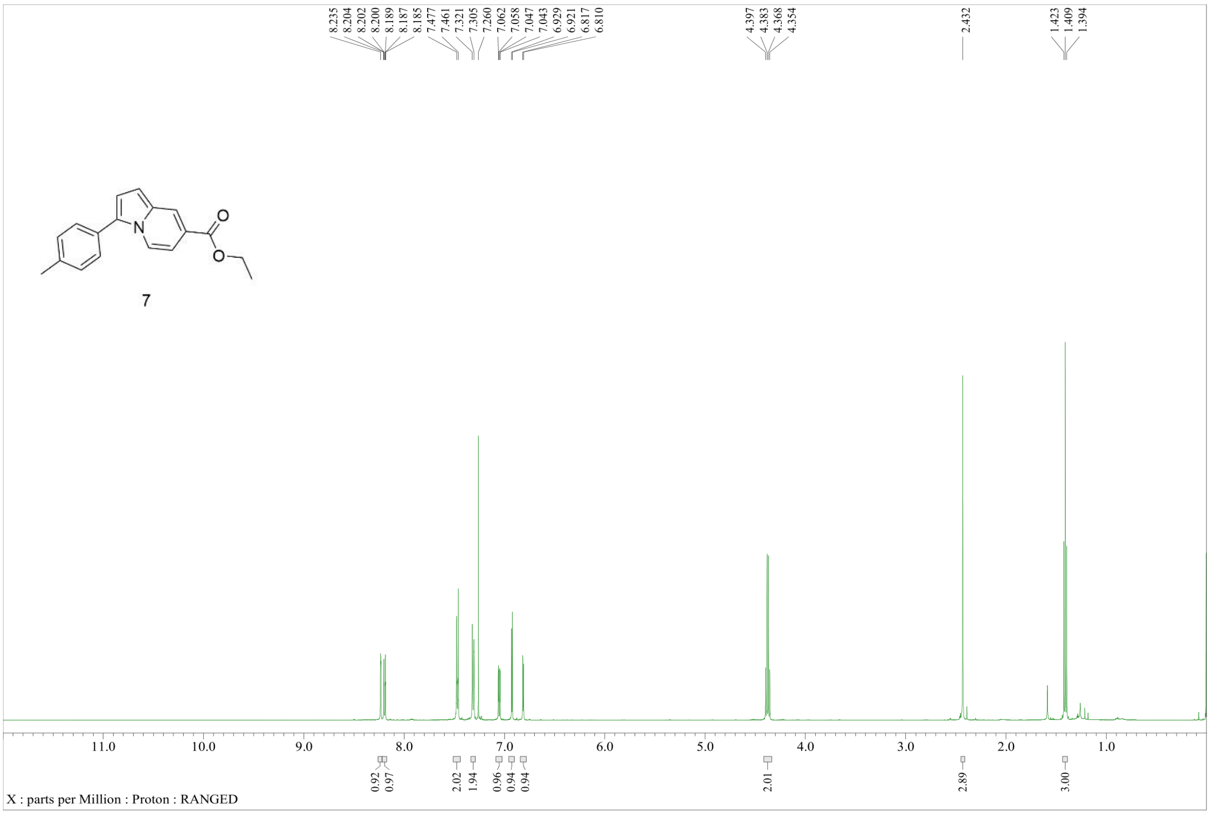
8.235 8.204 8.202 8.200 8.189 8.187 8.185 7.477 7.461 7.321 7.305 7.260 7.062 7.058 7.047 7.043 6.929 6.921 6.817 6.810
4.397 4.383 4.368 4.354
2.432
1.423 1.409 1.394
7
11.0 10.0 9.0 8.0 7.0 6.0 5.0 4.0 3.0 2.0 1.0
0.92 0.97 2.02 1.94 0.96 0.94 0.94 2.01 2.89 3.00
X : parts per Million : Proton : RANGED

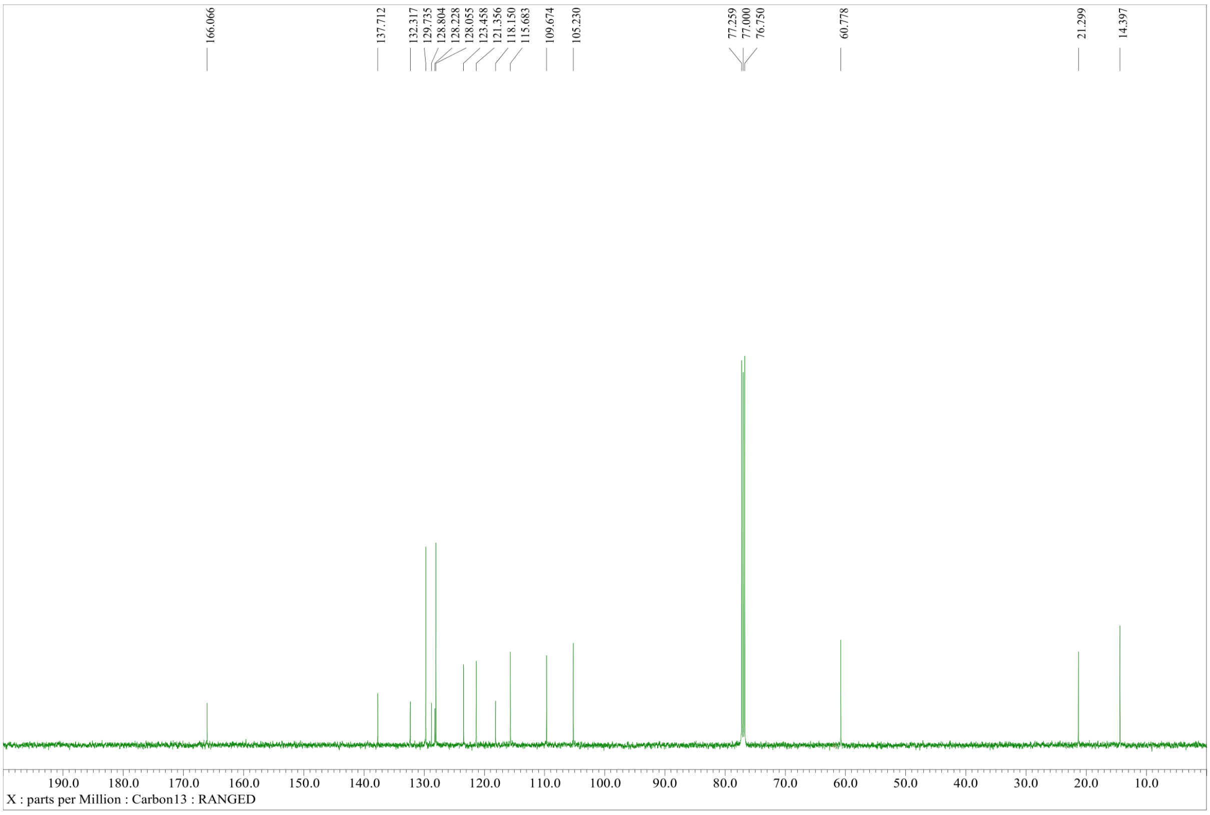
166.066
137.712 132.317 129.735 128.804 128.228 128.055 123.458 121.356 118.150 115.683 109.674 105.230
77.259 77.000 76.750
60.778
21.299 14.397
190.0 180.0 170.0 160.0 150.0 140.0 130.0 120.0 110.0 100.0 90.0 80.0 70.0 60.0 50.0 40.0 30.0 20.0 10.0
X : parts per Million : Carbon13 : RANGED

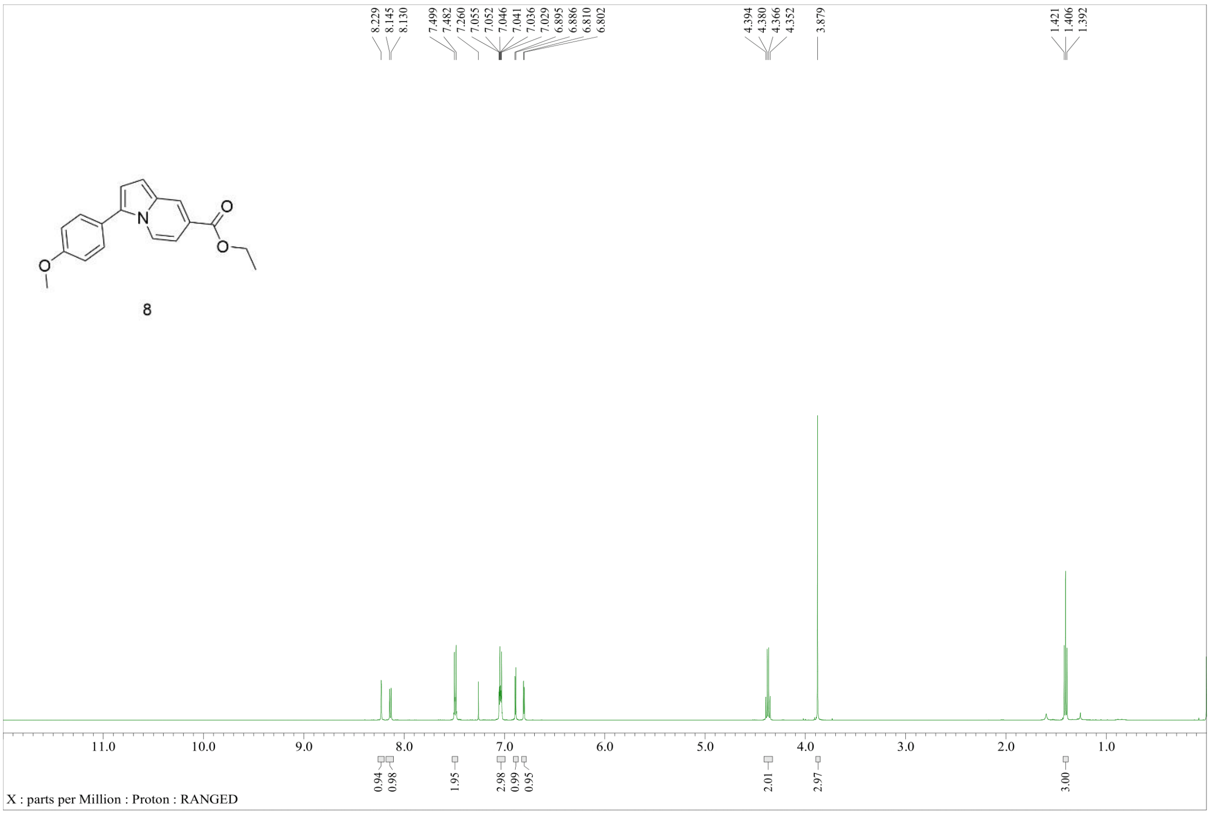
8.229 8.145 8.130 7.499 7.482 7.260 7.055 7.052 7.046 7.041 7.036 7.029 6.895 6.886 6.810 6.802
4.394 4.380 4.366 4.352 3.879
1.421 1.406 1.392
8
11.0 10.0 9.0 8.0 7.0 6.0 5.0 4.0 3.0 2.0 1.0
0.94 0.98 1.95 2.98 0.99 0.95 2.01 2.97 3.00
X : parts per Million : Proton : RANGED

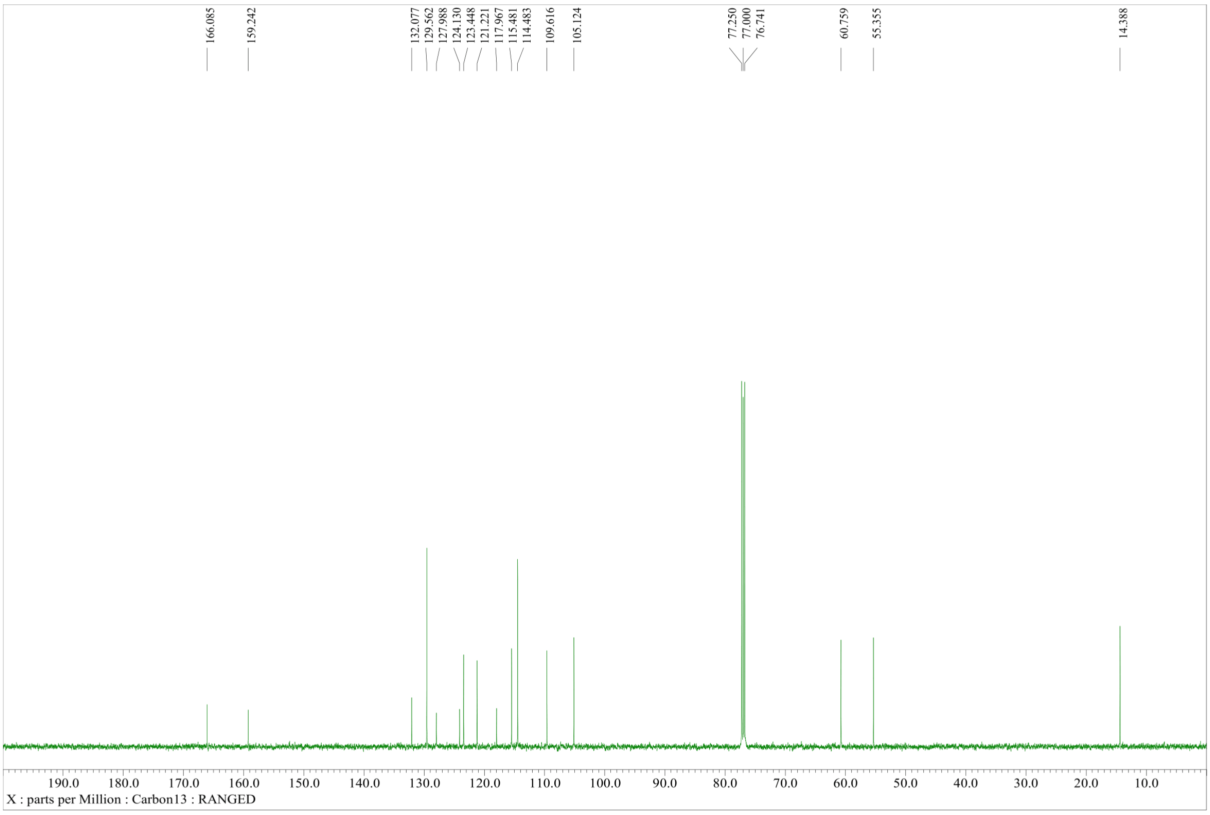
166.085 159.242
132.077 129.562 127.988 124.130 123.448 121.221 117.967 115.481 114.483 109.616 105.124
77.250 77.000 76.741
60.759 55.355 14.388
190.0 180.0 170.0 160.0 150.0 140.0 130.0 120.0 110.0 100.0 90.0 80.0 70.0 60.0 50.0 40.0 30.0 20.0 10.0
X : parts per Million : Carbon13 : RANGED

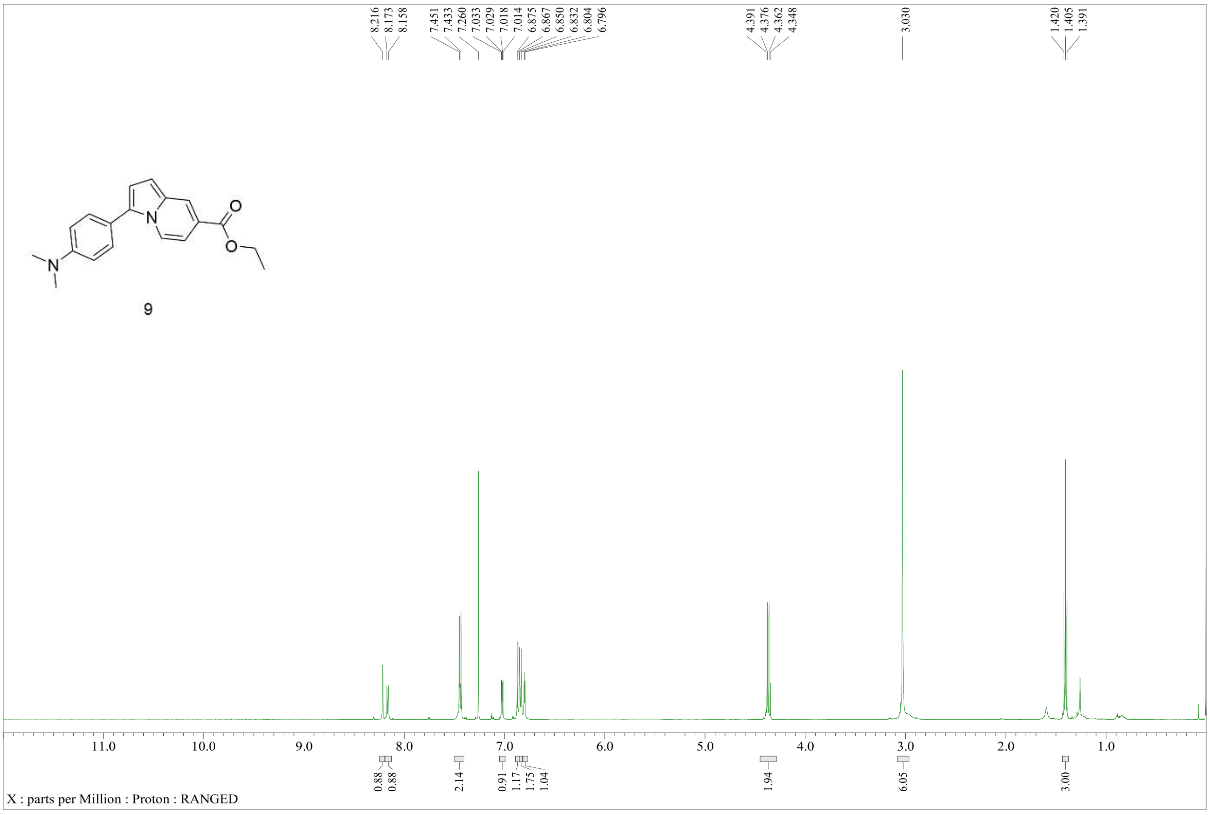
8.216
8.173
8.158
7.451
7.433
7.260
7.033
7.029
7.018
7.014
6.875
6.867
6.850
6.832
6.804
6.796
4.391
4.376
4.362
4.348
3.030
1.420
1.405
1.391
9
11.0 10.0 9.0 8.0 7.0 6.0 5.0 4.0 3.0 2.0 1.0
0.88
0.88
2.14
0.91
1.17
1.75
1.04
1.94
6.05
3.00
X : parts per Million : Proton : RANGED

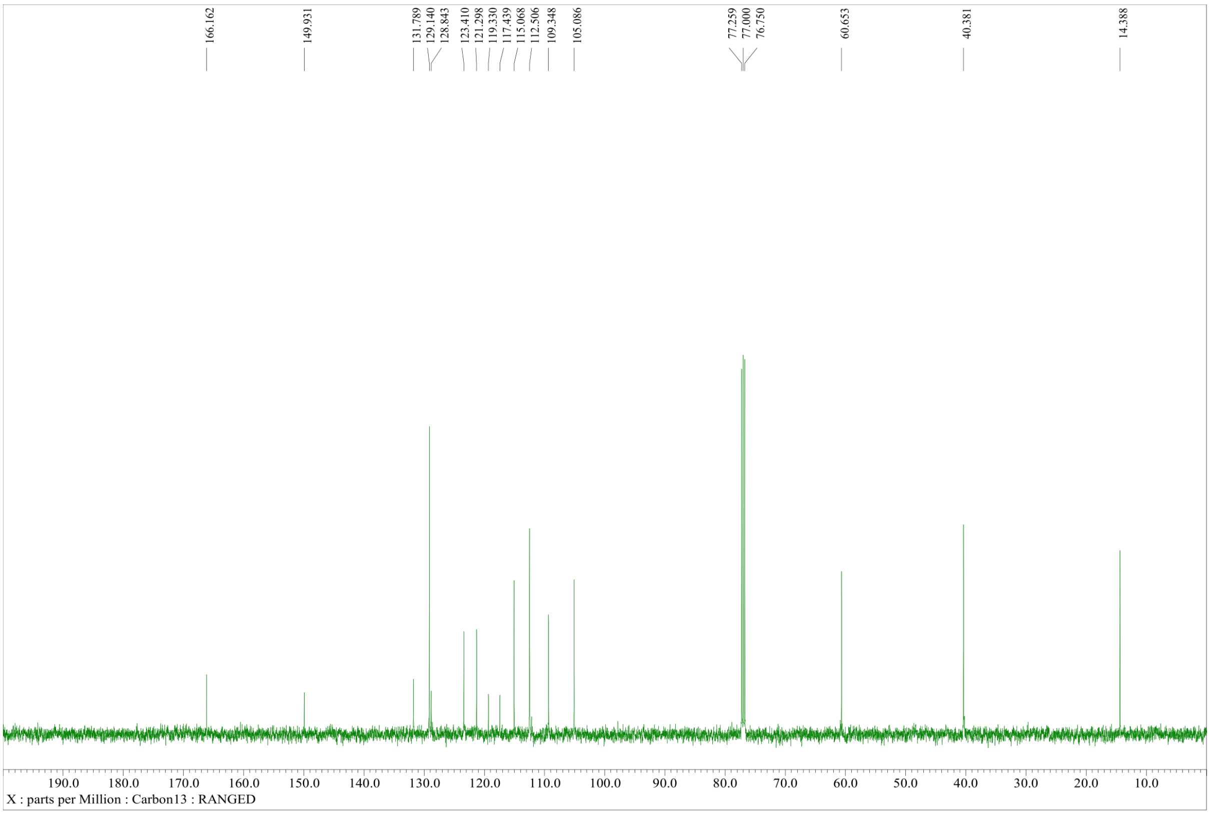
166.162
149.931
131.789
129.140
128.843
123.410
121.298
119.330
117.439
115.068
112.506
109.348
105.086
77.259
77.000
76.750
60.653
40.381
14.388
190.0 180.0 170.0 160.0 150.0 140.0 130.0 120.0 110.0 100.0 90.0 80.0 70.0 60.0 50.0 40.0 30.0 20.0 10.0
X : parts per Million : Carbon13 : RANGED

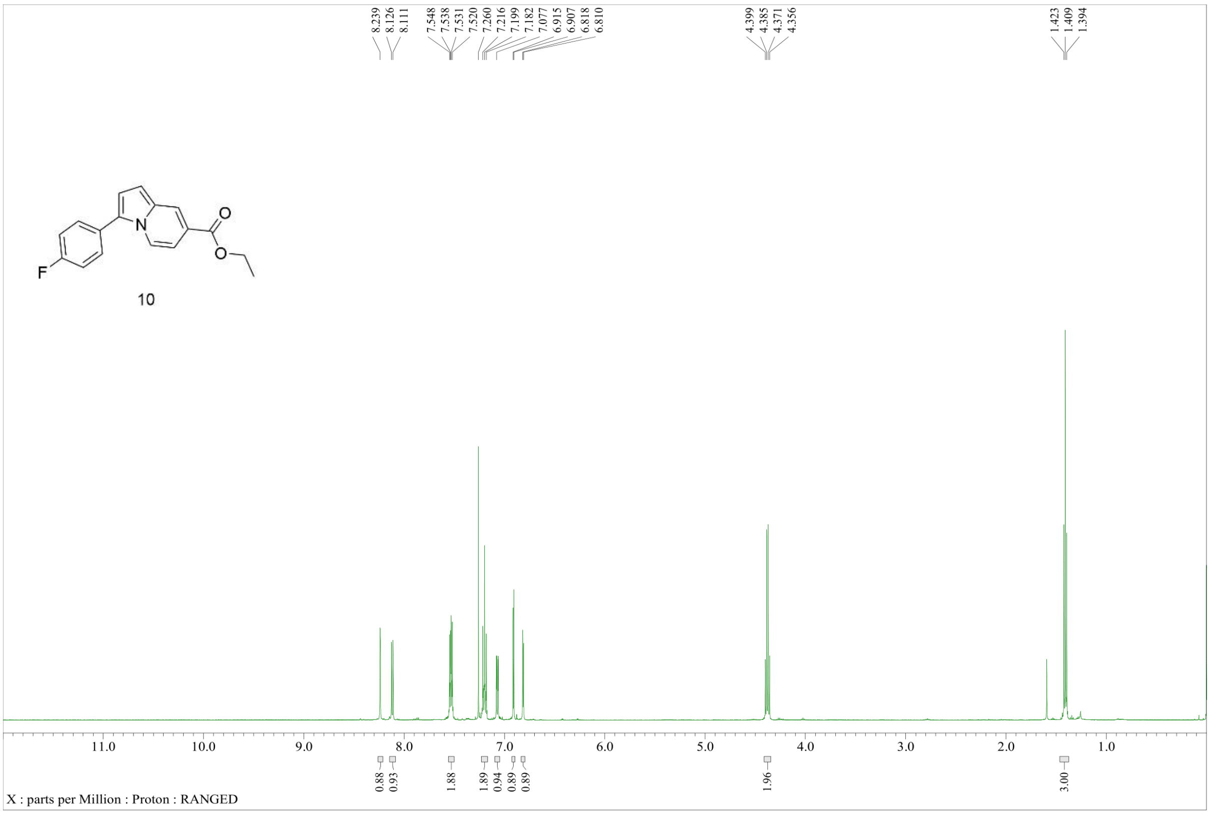
8.239
8.126
8.111
7.548
7.538
7.531
7.520
7.260
7.216
7.199
7.182
7.077
6.915
6.907
6.818
6.810
4.399
4.385
4.371
4.356
1.423
1.409
1.394
F
N
O
O
10
11.0
10.0
9.0
8.0
7.0
6.0
5.0
4.0
3.0
2.0
1.0
0.88
0.93
1.88
1.89
0.94
0.89
0.89
1.96
3.00
X : parts per Million : Proton : RANGED

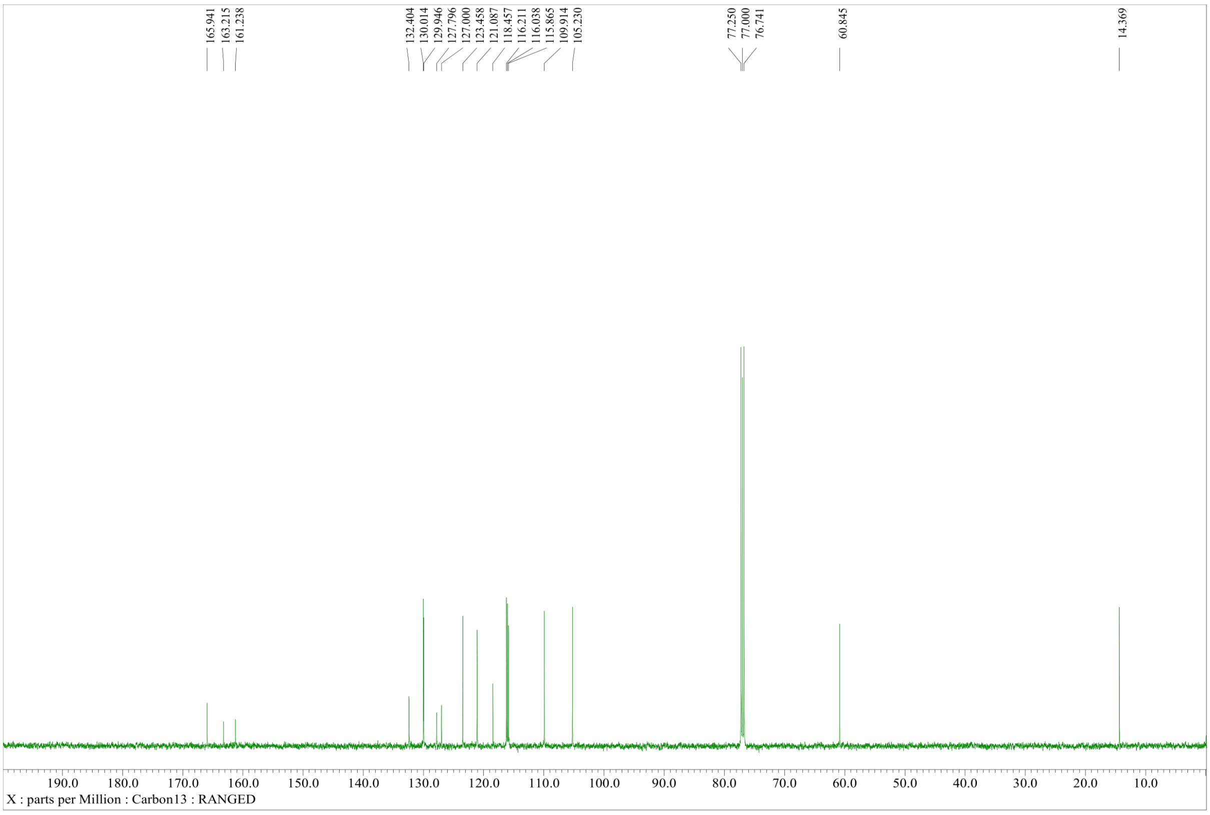
165.941
163.215
161.238
132.404
130.014
129.946
127.796
127.000
123.458
121.087
118.457
116.211
116.038
115.865
109.914
105.230
77.250
77.000
76.741
60.845
14.369
190.0
180.0
170.0
160.0
150.0
140.0
130.0
120.0
110.0
100.0
90.0
80.0
70.0
60.0
50.0
40.0
30.0
20.0
10.0
X : parts per Million : Carbon13 : RANGED

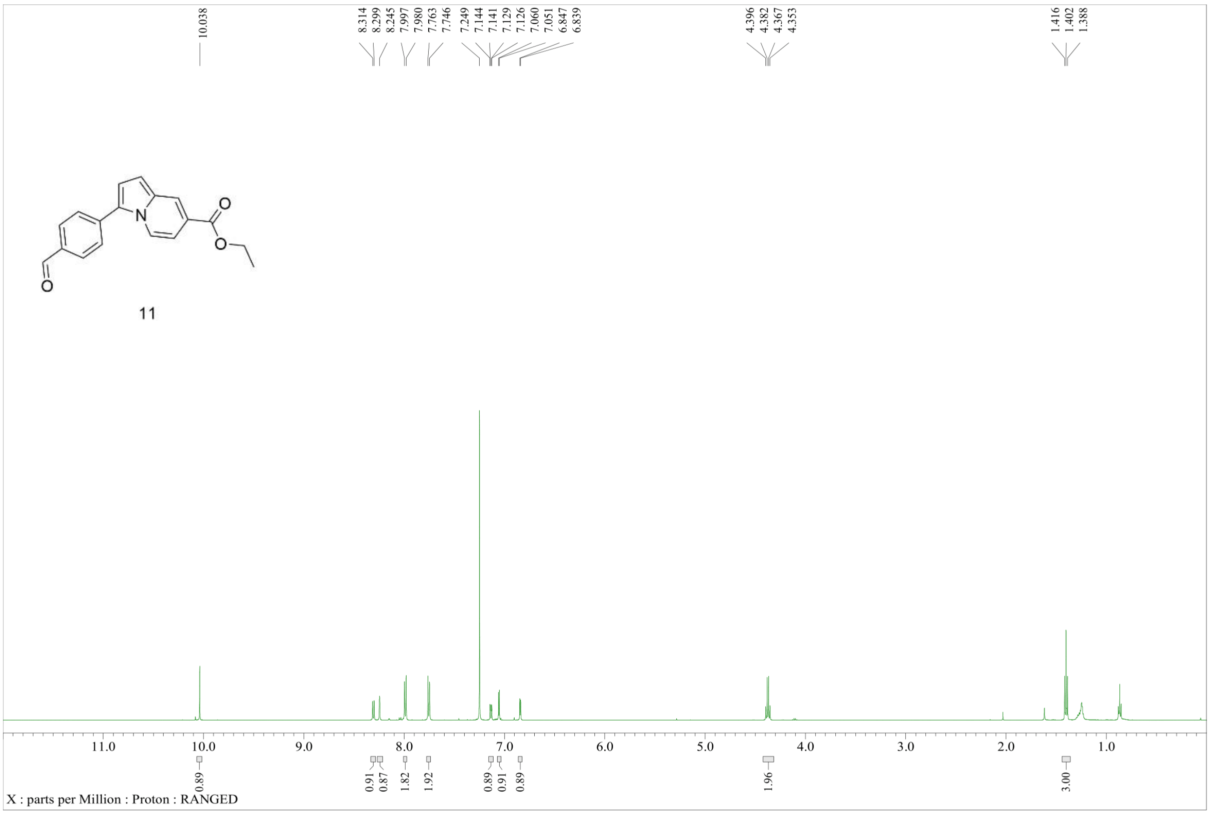

10.038
8.314
8.299
8.245
7.997
7.980
7.763
7.746
7.249
7.144
7.141
7.129
7.126
7.060
7.051
6.847
6.839
4.396
4.382
4.367
4.353
1.416
1.402
1.388
11
0.89
0.91
0.87
1.82
1.92
0.89
0.91
0.89
1.96
3.00
X : parts per Million : Proton : RANGED

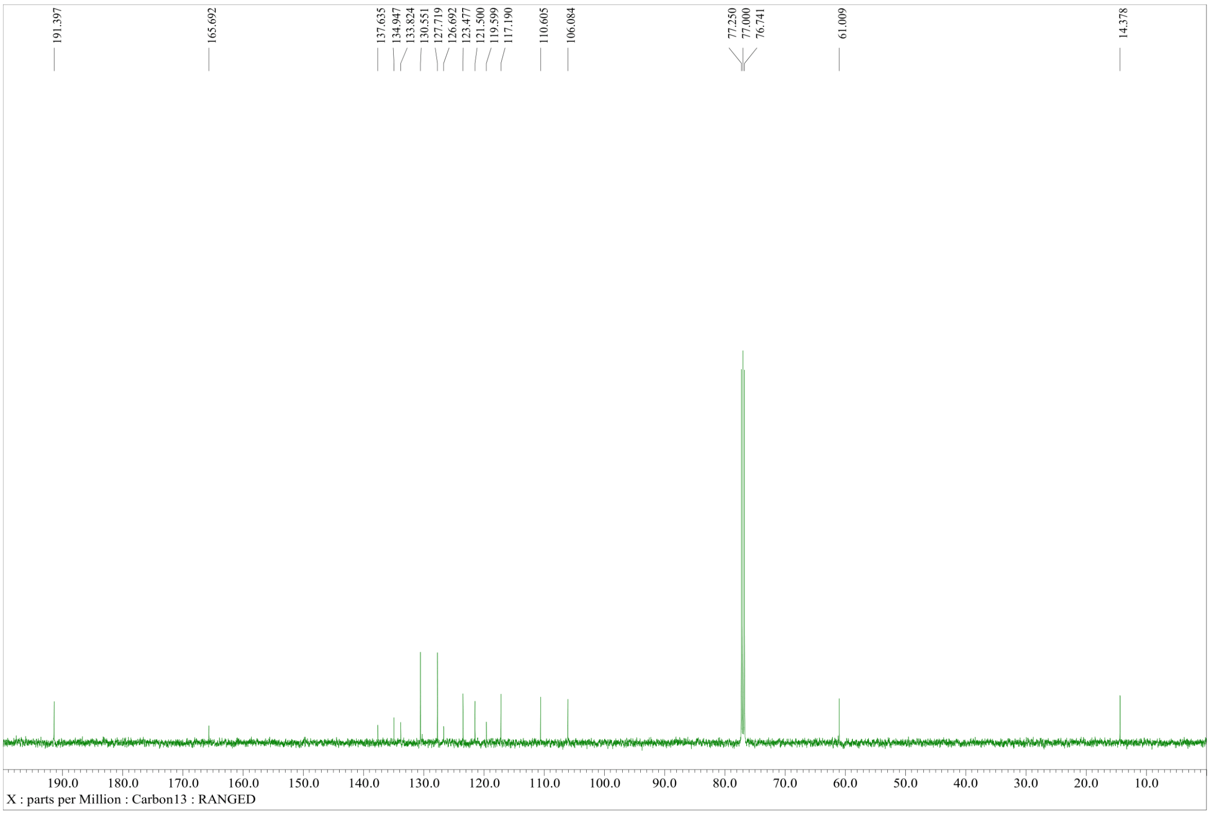

191.397
165.692
137.635
134.947
133.824
130.551
127.719
126.692
123.477
121.500
119.599
117.190
110.605
106.084
77.250
77.000
76.741
61.009
14.378
X : parts per Million : Carbon13 : RANGED

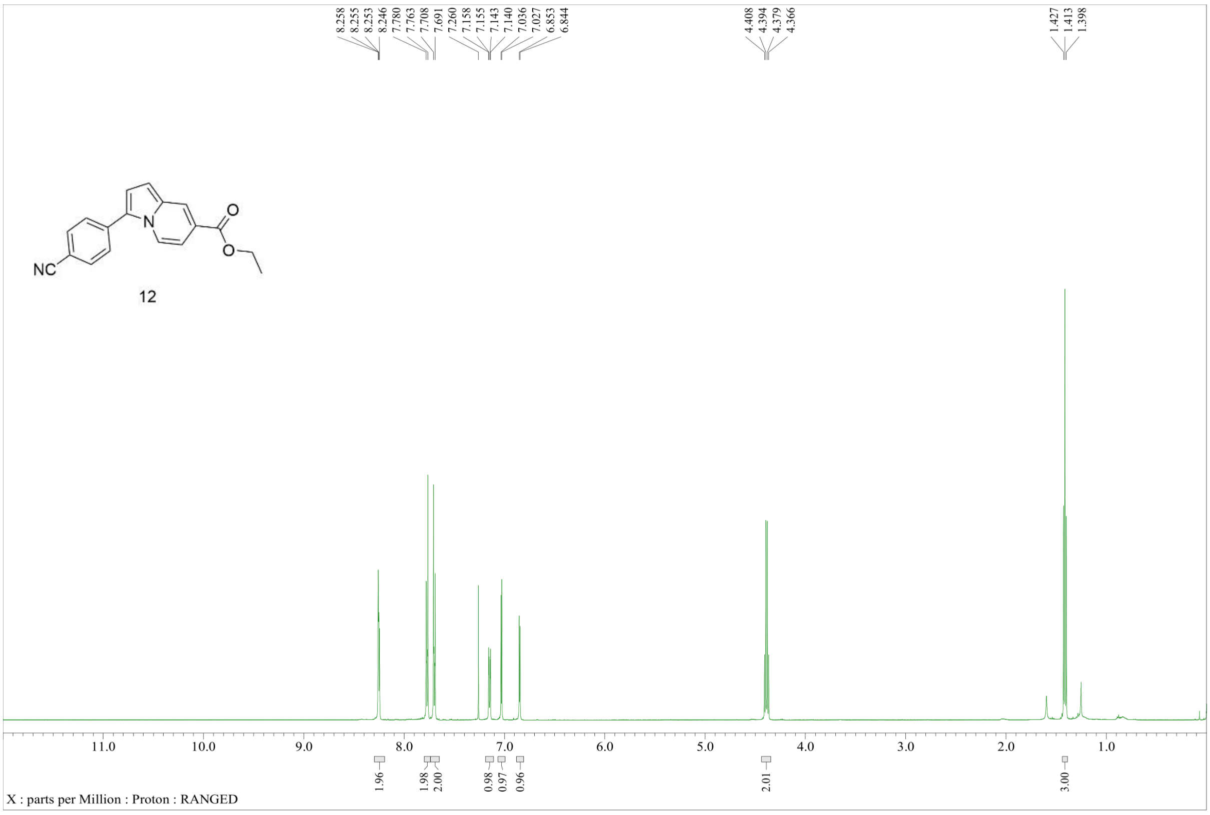
8.258
8.255
8.253
8.246
7.780
7.763
7.708
7.691
7.260
7.158
7.155
7.143
7.140
7.036
7.027
6.853
6.844
4.408
4.394
4.379
4.366
1.427
1.413
1.398
NC
O
N
O
12
1.96
1.98
2.00
0.98
0.97
0.96
2.01
3.00
X : parts per Million : Proton : RANGED

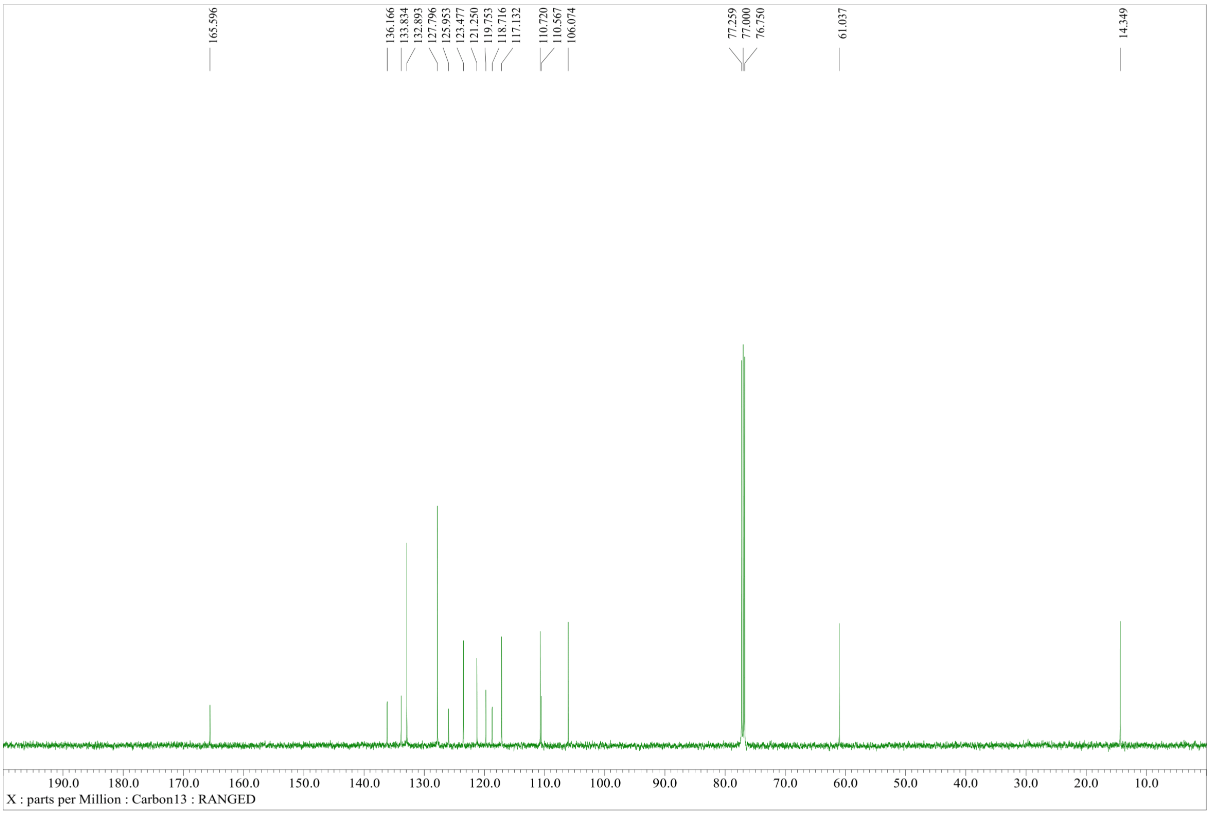
165.596
136.166
133.834
132.893
127.796
125.953
123.477
121.250
119.753
118.716
117.132
110.720
110.567
106.074
77.259
77.000
76.750
61.037
14.349
X : parts per Million : Carbon13 : RANGED

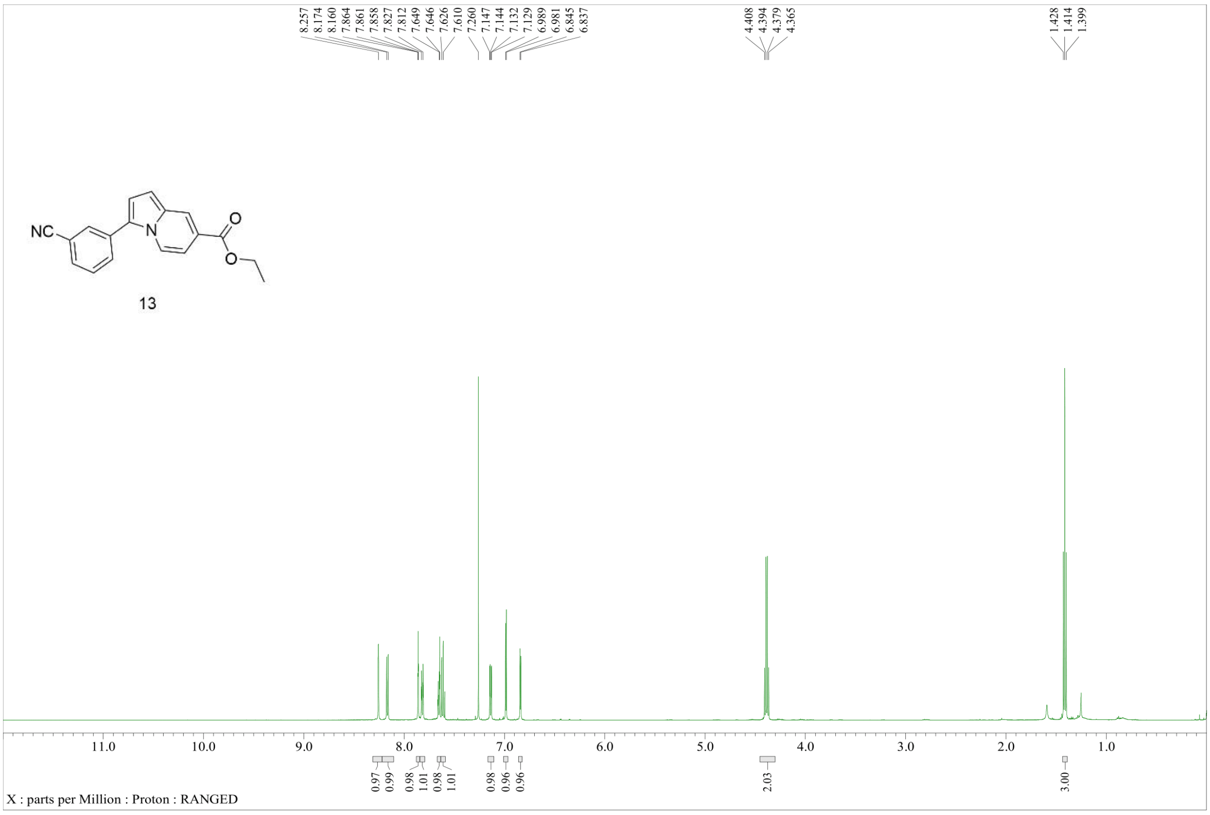

8.257
8.174
8.160
7.864
7.861
7.858
7.827
7.812
7.649
7.646
7.626
7.610
7.260
7.147
7.144
7.132
7.129
6.989
6.981
6.845
6.837
4.408
4.394
4.379
4.365
1.428
1.414
1.399
NC
N
O
O
13
11.0
10.0
9.0
8.0
7.0
6.0
5.0
4.0
3.0
2.0
1.0
0.97
0.99
0.98
1.01
0.98
1.01
0.98
0.96
0.96
2.03
3.00
X : parts per Million : Proton : RANGED

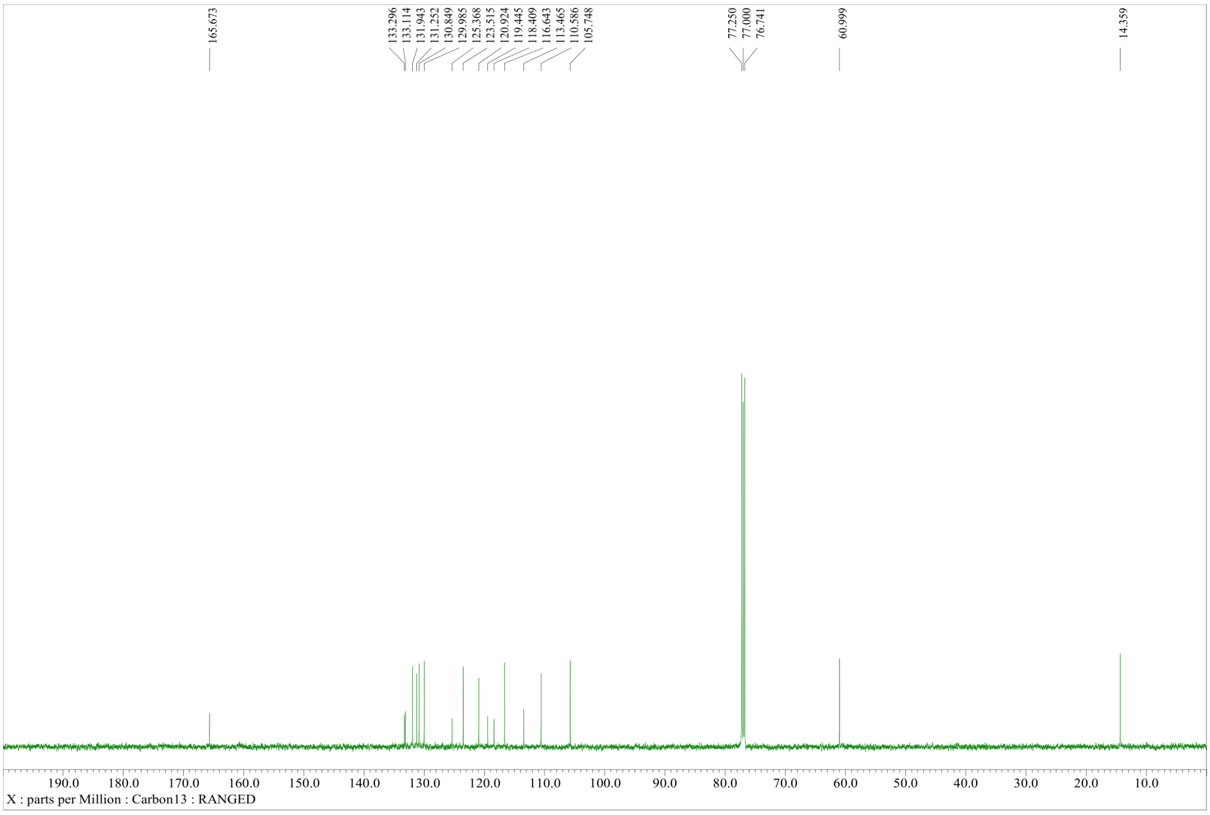

165.673
133.296
133.114
131.943
131.252
130.849
129.985
125.368
123.515
120.924
119.445
118.409
116.643
113.465
110.586
105.748
77.250
77.000
76.741
60.999
14.359
190.0
180.0
170.0
160.0
150.0
140.0
130.0
120.0
110.0
100.0
90.0
80.0
70.0
60.0
50.0
40.0
30.0
20.0
10.0
X : parts per Million : Carbon13 : RANGED

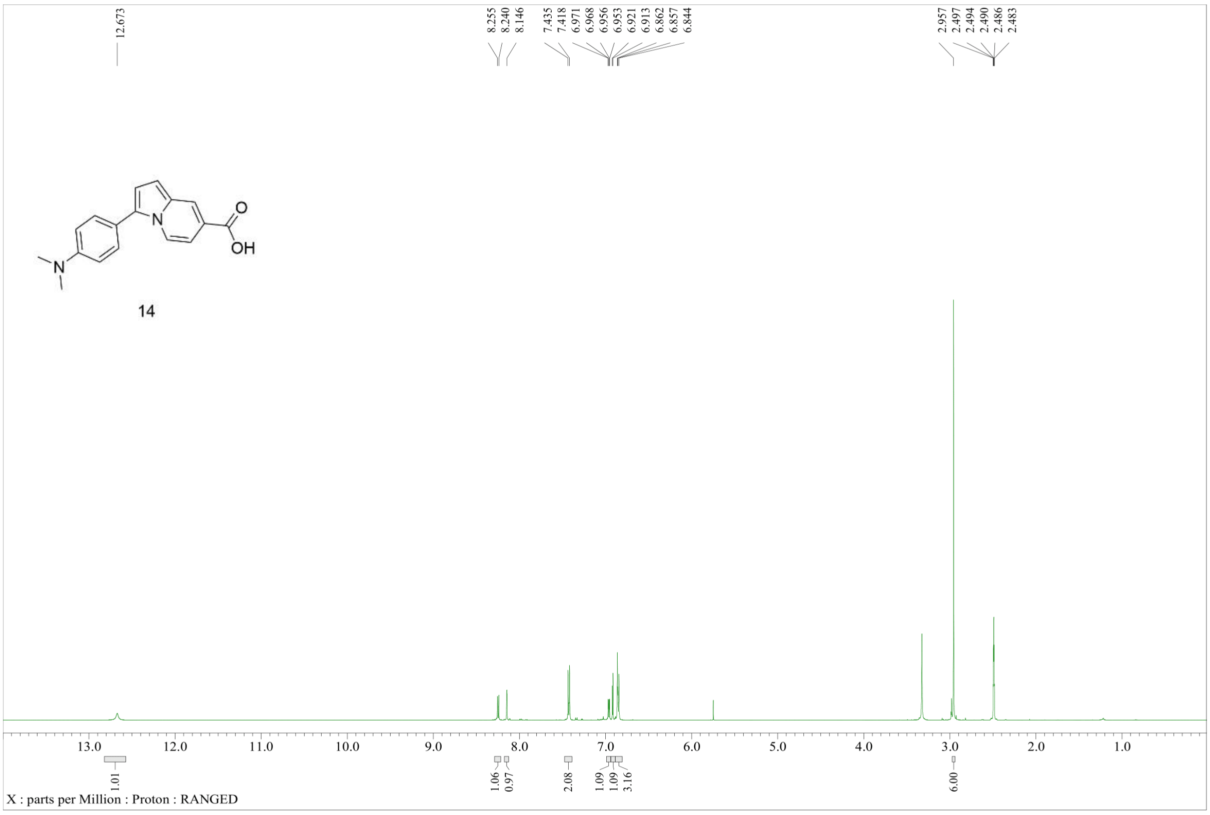
12.673
8.255
8.240
8.146
7.435
7.418
6.971
6.968
6.956
6.953
6.921
6.913
6.862
6.857
6.844
2.957
2.497
2.494
2.490
2.486
2.483
O
OH
N
N
14
13.0
12.0
11.0
10.0
9.0
8.0
7.0
6.0
5.0
4.0
3.0
2.0
1.0
1.01
1.06
0.97
2.08
1.09
1.09
3.16
6.00
X : parts per Million : Proton : RANGED

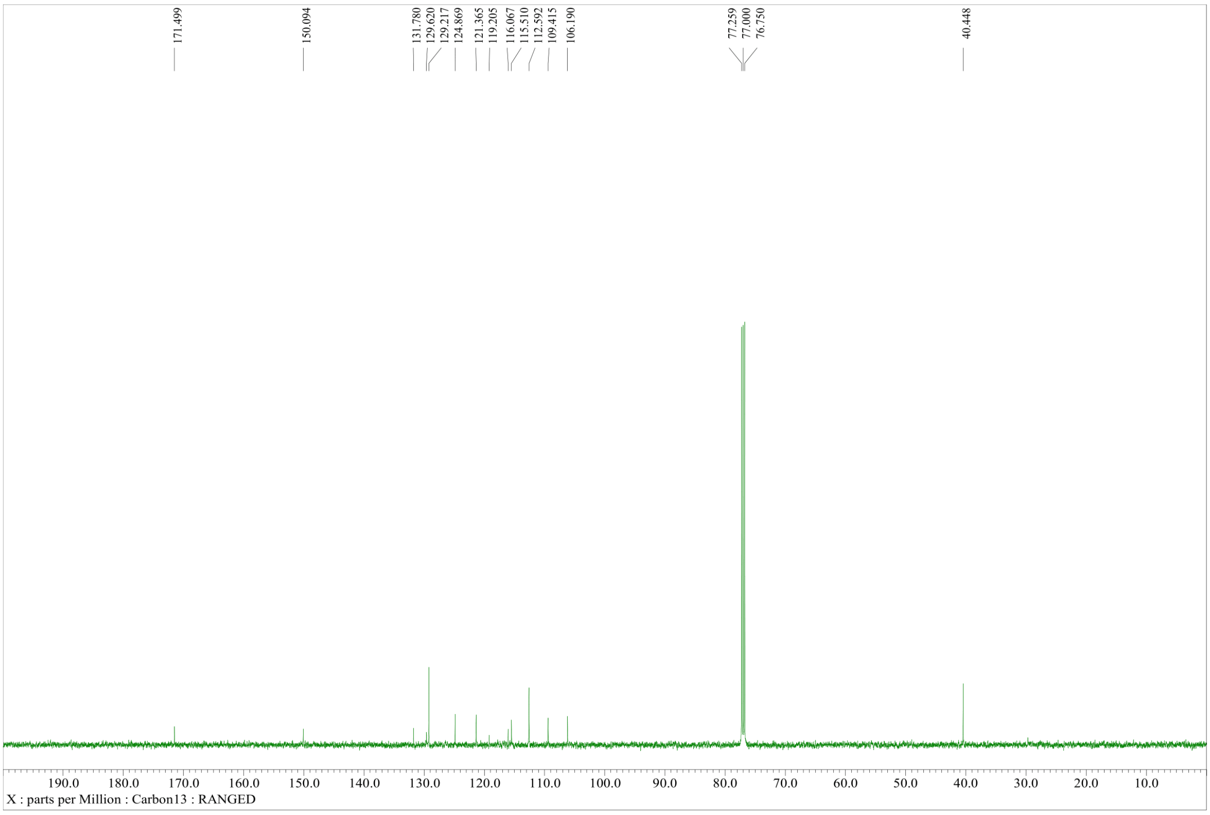
171.499
150.094
131.780
129.620
129.217
124.869
121.365
119.205
116.067
115.510
112.592
109.415
106.190
77.259
77.000
76.750
40.448
190.0
180.0
170.0
160.0
150.0
140.0
130.0
120.0
110.0
100.0
90.0
80.0
70.0
60.0
50.0
40.0
30.0
20.0
10.0
X : parts per Million : Carbon13 : RANGED

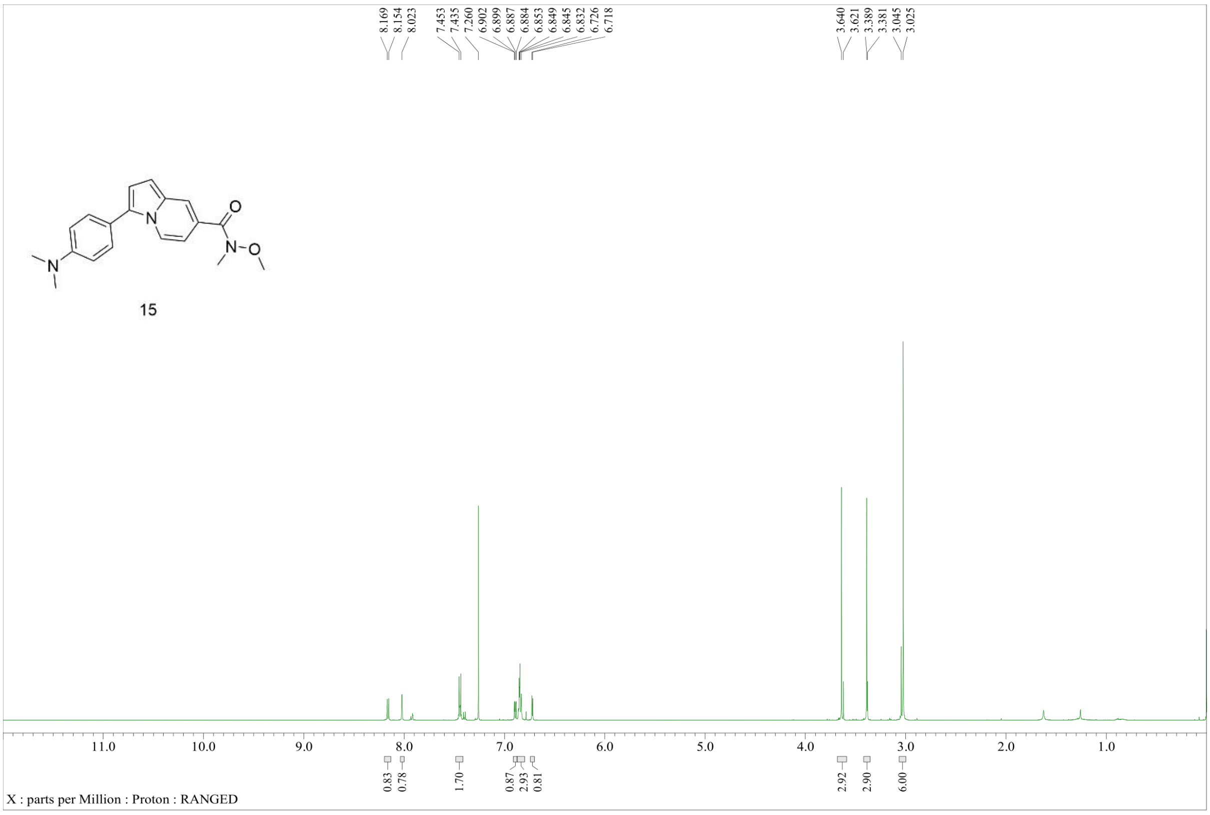
8.169
8.154
8.023
7.453
7.435
7.260
6.902
6.899
6.887
6.884
6.853
6.849
6.845
6.832
6.726
6.718
3.640
3.621
3.389
3.381
3.045
3.025
N
O
N
O
N
15
11.0
10.0
9.0
8.0
7.0
6.0
5.0
4.0
3.0
2.0
1.0
0.83
0.78
1.70
0.87
2.93
0.81
2.92
2.90
6.00
X : parts per Million : Proton : RANGED

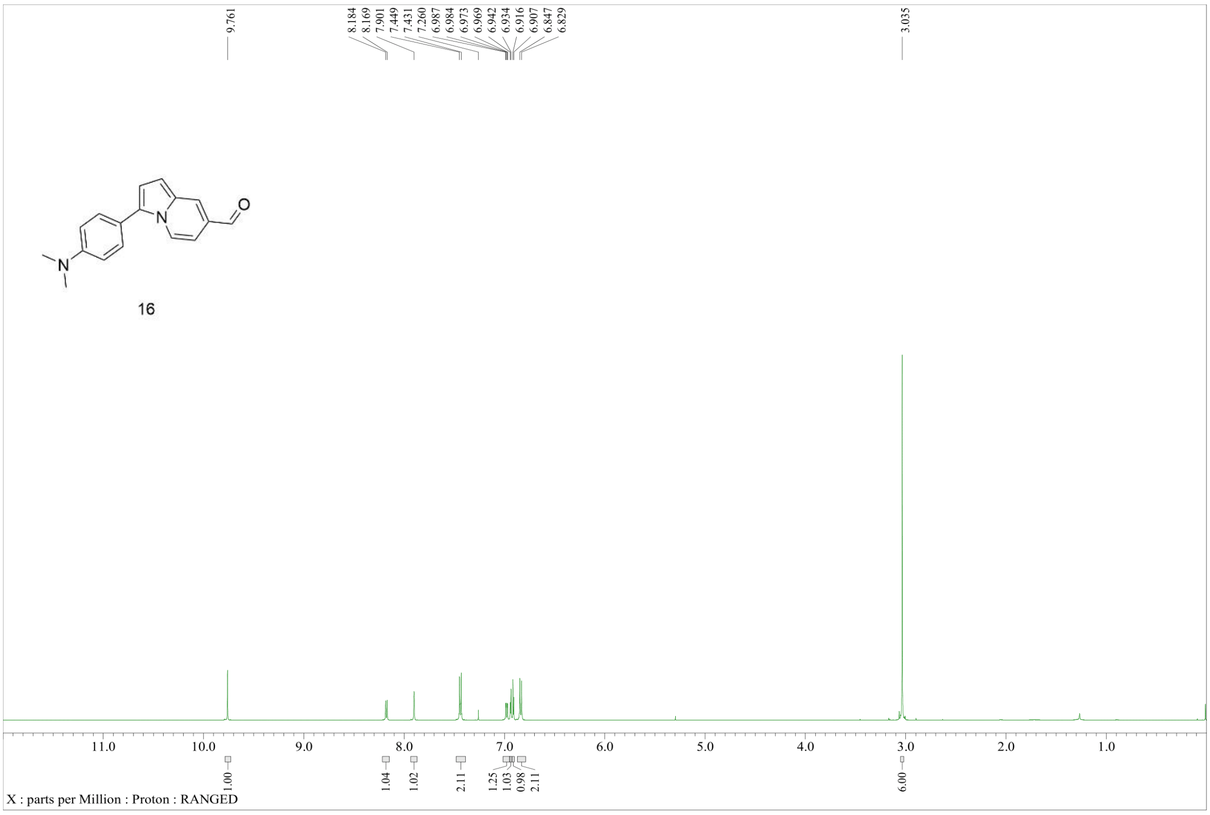
9.761
8.184
8.169
7.901
7.449
7.431
7.260
6.987
6.984
6.973
6.969
6.942
6.934
6.916
6.907
6.847
6.829
3.035
O
N
N
16
11.0 10.0 9.0 8.0 7.0 6.0 5.0 4.0 3.0 2.0 1.0
1.00
1.04
1.02
2.11
1.25
1.03
0.98
2.11
6.00
X : parts per Million : Proton : RANGED

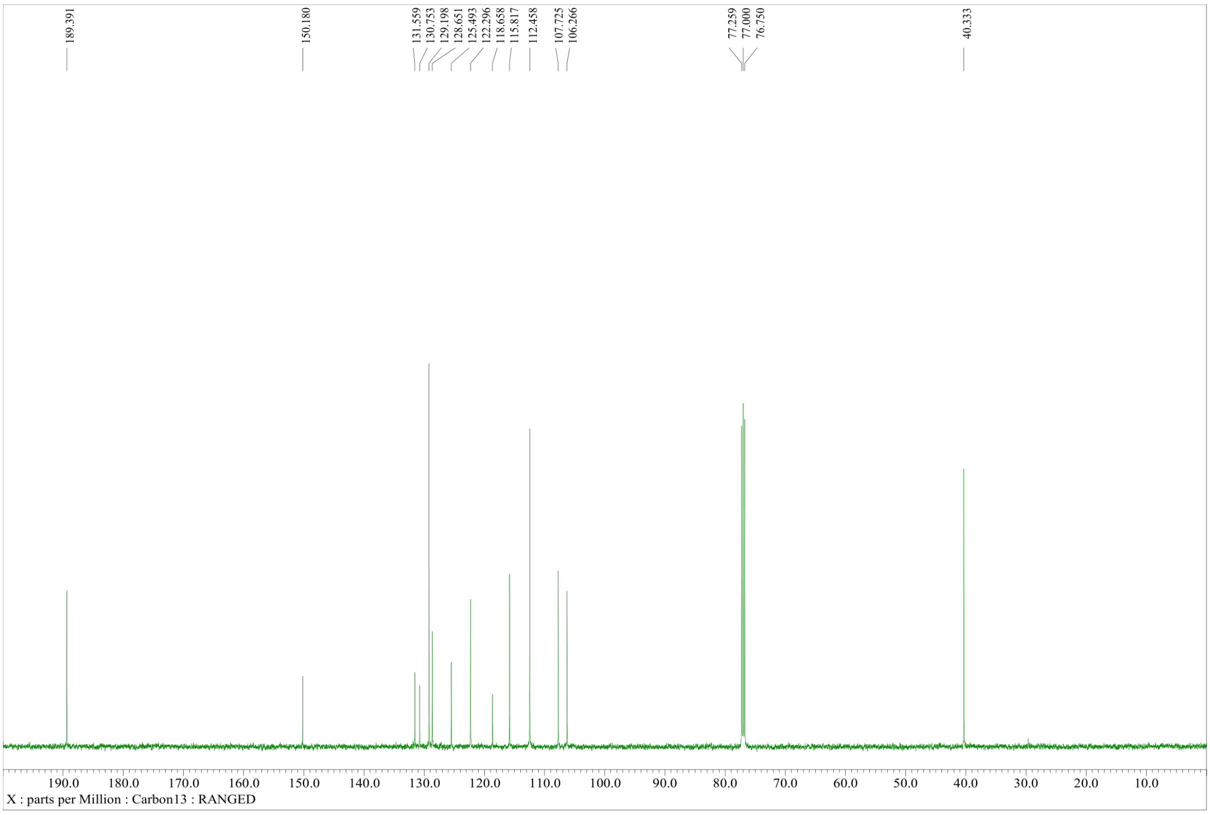
189.391
150.180
131.559
130.753
129.198
128.651
125.493
122.296
118.658
115.817
112.458
107.725
106.266
77.259
77.000
76.750
40.333
190.0 180.0 170.0 160.0 150.0 140.0 130.0 120.0 110.0 100.0 90.0 80.0 70.0 60.0 50.0 40.0 30.0 20.0 10.0
X : parts per Million : Carbon13 : RANGED

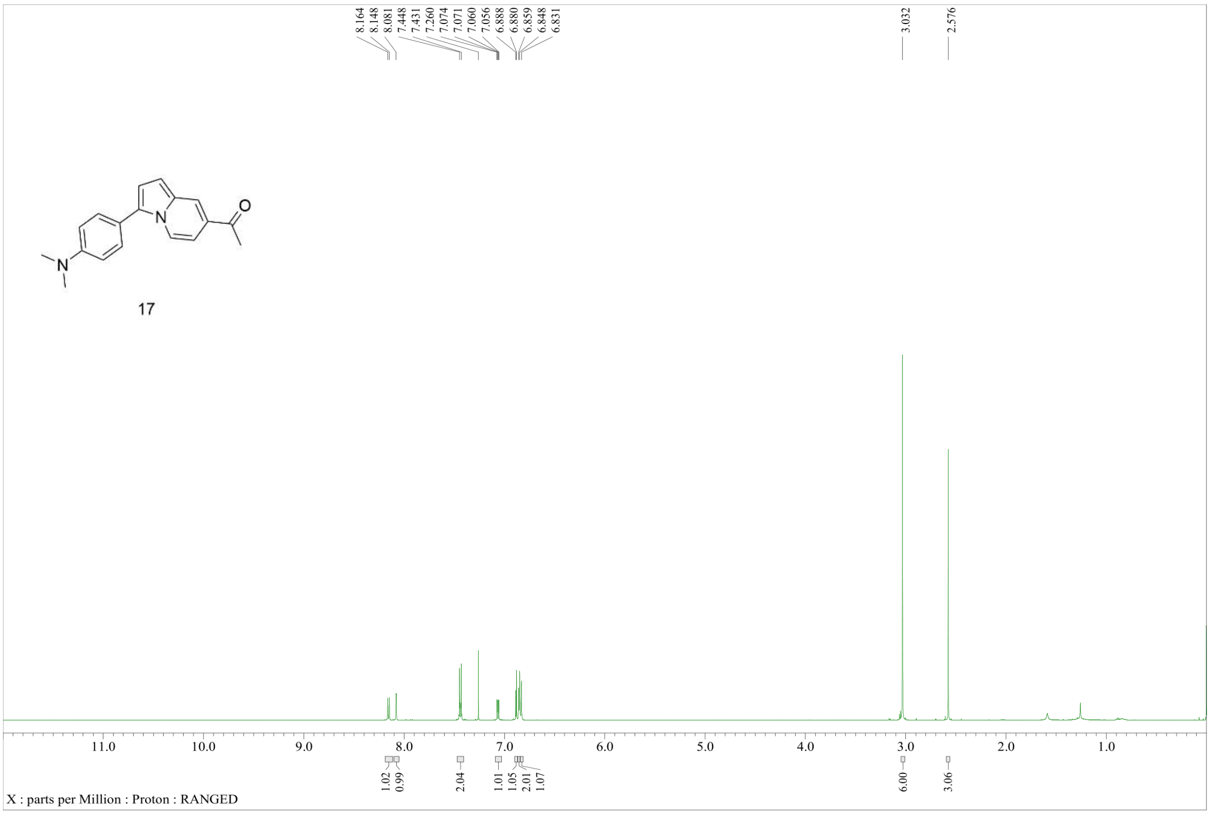
8.164
8.148
8.081
7.448
7.431
7.260
7.074
7.071
7.060
7.056
6.888
6.880
6.859
6.848
6.831
3.032
2.576
17
1.02
0.99
2.04
1.01
1.05
2.01
1.07
6.00
3.06
11.0
10.0
9.0
8.0
7.0
6.0
5.0
4.0
3.0
2.0
1.0
X : parts per Million : Proton : RANGED

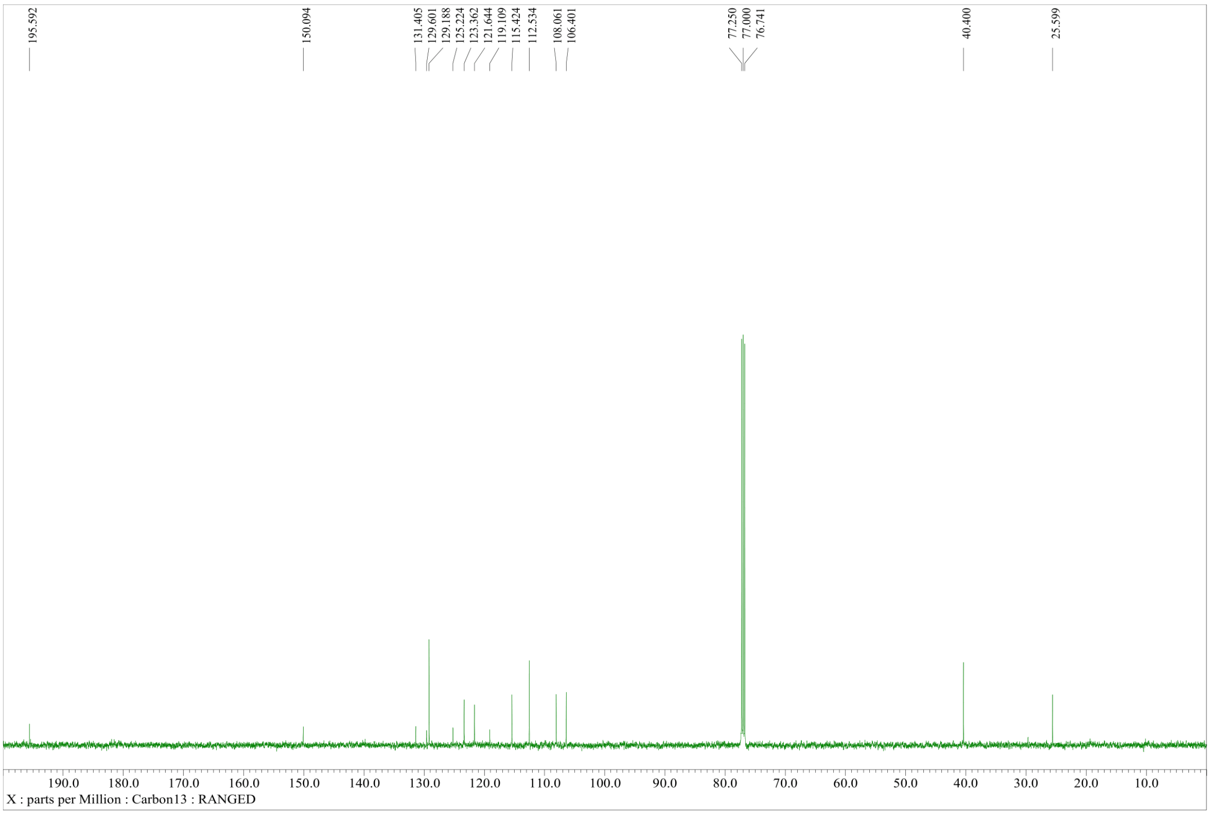
195.592
150.094
131.405
129.601
129.188
125.224
123.362
121.644
119.109
115.424
112.534
108.061
106.401
77.250
77.000
76.741
40.400
25.599
190.0
180.0
170.0
160.0
150.0
140.0
130.0
120.0
110.0
100.0
90.0
80.0
70.0
60.0
50.0
40.0
30.0
20.0
10.0
X : parts per Million : Carbon13 : RANGED
